# Quantifying spectral information and redundancy in high-dimensional personality taxonomies: an entropy-based approach

**DOI:** 10.3389/fpsyg.2026.1820233

**Published:** 2026-07-29

**Authors:** Fernando Gutiérrez, Anton Aluja, José Ruiz-Rodríguez, Luis F. García, Eva Baillès, Natalia Calvo, Claudia Rodríguez, Miguel Gárriz

**Affiliations:** 1 Institute of Neuroscience, Hospital Clínic of Barcelona, Barcelona, Spain; 2 Institut d’Investigacións Biomèdiques August Pi i Sunyer (IDIBAPS), Barcelona, Spain; 3 Lleida Institute for Biomedical Research Dr. Pifarré Foundation, Lleida, Spain; 4 Department of Clinical Psychology and Psychobiology, University of Barcelona (UB), Barcelona, Spain; 5 Institute of Neuroscience (UBneuro), University of Barcelona (UB), Barcelona, Spain; 6 Department of Biological and Health Psychology, Universidad Autónoma de Madrid, Madrid, Spain; 7 Consultation Liaison Psychiatry Section, Department of Psychiatry, Vall d'Hebron University Hospital, Barcelona, Spain; 8 Department of Psychiatry, Vall d’Hebron University Hospital, CIBERSAM, Barcelona, Spain; 9 Psychiatry, Mental Health, and Addictions Group, Vall d’Hebron Research Institute (VHIR), UAB, Barcelona, Spain

**Keywords:** curse of dimensionality, effective dimensionality, personality assessment, personality taxonomy, Shannon entropy, spectral information

## Abstract

**Background:**

Personality assessment involves the collection and organization of information into multidimensional taxonomies. However, no routinely used index directly quantifies the amount of information encoded in such taxonomies. Effective dimensionality (ED) is a Shannon entropy-based metric that uses eigenvalue distributions to quantify a system’s spectral information as the effective number of non-redundant components. Although ED has been applied across fields ranging from physics to political science, it remains underutilized in psychological assessment.

**Methods:**

After establishing an interpretive framework through Monte Carlo simulation, we applied ED to quantify informational content and redundancy across five widely used taxonomies of normal and pathological personality in large community and clinical samples (*n* = 2,510–7,953), as well as across hierarchical levels of measurement (domains, facets, items), alternative factor-analytic solutions, and populations.

**Results:**

On average, domains conveyed 21% less spectral information than their nominal dimensionality implies, and facets 45% less, reflecting substantial trait overlap. Across levels, items contained 9- to 31-fold greater spectral information than domains, reflecting both specific variance and measurement error. Although residualized facets and items contained unique variance that predicted clinically relevant life outcomes, gains did not reach significance under stringent out-of-sample comparisons. In factor analysis, ED quantified factor redundancy across rotation methods, with oblique rotations producing up to 25% less spectral information. ED also informed cutoff calibration to mitigate the curse of dimensionality—which can artifactually inflate disorder rates in high-dimensional systems—maintaining prevalence estimates within 1–15% of target levels under multivariate normality.

**Conclusion:**

ED provides a model-agnostic, continuous index for quantifying spectral information, structural redundancy, and complexity in multidimensional psychological systems. However, it does not necessarily coincide with psychologically meaningful information. ED may support the development of informationally parsimonious taxonomies and well-calibrated diagnostic thresholds in high-dimensional personality assessment.

## 1 Introduction

The currency of personality assessment is information. Psychometric instruments collect extensive data on patients’ stable inner experiences and overt behaviors, organizing them into meaningful taxonomies that guide diagnosis and treatment. Yet a simple, general metric to quantify the information contained in these taxonomies has been lacking (Del Giudice, 2021).

Effective dimensionality (ED) is a candidate for such a metric. ED applies spectral entropy to the normalized eigenvalues of a covariance or correlation matrix to estimate the effective number of equal-sized orthogonal dimensions that capture the variance structure of a set of variables (Del Giudice, 2021; Gnedenko and Yelnik, 2016). Insofar as personality traits correlate, they can be expressed as a smaller number of independent dimensions. For example, if the five domains of the Alternative Model of Personality Disorders (AMPD; American Psychiatric Association, 2013)—negative emotionality, detachment, antagonism, disinhibition, and psychoticism—were perfectly correlated, their correlation matrix would yield an ED of 1. If the domains were completely orthogonal, ED would equal 5. In practice, however, the domains overlap partially, producing values between these extremes.

Effective dimensionality is grounded in information theory. Specifically, it draws from Shannon entropy (Shannon, 1948), which measures the uncertainty or randomness of a probability distribution, and thus its informational value (Stone, 2015). In probabilistic terms, uncertainty can be understood as the average minimum number of binary questions required to determine the outcome of an event. For example, only one question is needed to guess the outcome of a coin flip, but an average of 2.58 for a die roll (Cangelosi and Goriely, 2007). In essence, uncertainty captures the amount of surprise in the obtained information. By analogy, each personality trait in a taxonomy is expected to contribute additional non-redundant information after all other traits are known. Entropy thus captures the degree of diversification within a universe of variables: the more closely related the variables, the less they reduce uncertainty and the less diverse they are (Budescu and Budescu, 2012; Pirkl et al., 2012). In the present context, entropy is applied not to the raw data but to the normalized eigenvalue spectrum of the covariance or correlation matrix. Accordingly, the term information refers throughout this paper to spectral information—that is, information about how variance is distributed across orthogonal dimensions, reflecting the meaningful, non-redundant signal in the system.

Shannon entropy (*H*) provides a unified framework for quantifying spectral information, uncertainty, and diversity—or complexity—across multiple fields. It has been used in ecology to determine the phylogenetic diversity of species (Kosman et al., 2024), in population genetics to analyze the dimensionality of the genetic variance–covariance matrix G (Sherwin et al., 2017), in physics to measure disorder in quantum systems (Preskill, 2018), in economics as an indicator of systemic risk in financial markets (Fleming and Kroeske, 2013), and in political science to measure the effective number of political parties (Laakso and Taagepera, 1979). Depending on the discipline, closely related formulations include the Hill number of order 1, effective rank, and von Neumann entropy (Cover and Thomas, 2006; Crooks, 2024; Roy and Vetterli, 2007; Verdú, 2019). Entropy-based metrics also have broad potential applications across psychological and psychopathological taxonomies. However, despite strong conceptual links (Budescu and Budescu, 2012; Condon and Mõttus, 2021), their use in personality assessment has remained limited.

We outline six substantive applications of ED in personality research. First, ED enables direct comparisons between instruments—and, by extension, their underlying taxonomies—in terms of the amount of spectral information they encode. While such comparisons are crucial for instrument selection, those based on nominal domain counts are misleading because domains are typically intercorrelated. For example, researchers may wish to determine whether two five-domain taxonomies, such as those proposed in the DSM-5 (American Psychiatric Association, 2013) and the ICD-11 (World Health Organization, 2018), are equally informative, or whether a broader seven-dimensional model—such as Cloninger’s biosocial model—exceeds both.

Second, the focus may be not on the absolute amount of information but on redundancy—the proportion of variance that contributes no new information (Stone, 2015). Overlapping constructs create conceptual ambiguity, complicate clinical decision-making, and hinder the identification of unshared causal mechanisms and consequences (Lahey, 2023). Indeed, a primary reason for discontinuing categorical diagnoses has been their high co-occurrence rates (Monaghan and Bizumic, 2023). The discrepancy between nominal and effective dimensionality quantifies this redundancy directly and enables evaluation of whether dimensional taxonomies have resolved the overlap issue. Likewise, ED can be computed for two or more systems jointly, offering insights into their degree of overlap, the incremental contribution of each, and their total informational content when combined.

Third, ED enables comparisons of personality structural complexity across populations. For instance, personality differences have been hypothesized to be more pronounced in developed societies than in pre-industrial ones, owing to greater niche diversity and role specialization (Durkee et al., 2022). Analogously, pathological traits are hypothesized to overlap more extensively than normal-range traits because they share a common underlying component of dysfunction (Morey et al., 2022). Although such proposals have typically been evaluated by inspecting correlation matrices, ED may offer a more parsimonious and interpretable metric (Del Giudice, 2021; Pirkl et al., 2012).

Fourth, ED enables comparisons across hierarchical levels within a taxonomy. The informational gain from using facets or items rather than domains has been recently debated (Mõttus et al., 2019). Although increasing the number of traits typically raises total information, the marginal contribution per trait decreases as dimensionality grows (Stone, 2015). Therefore, whether narrower, highly intercorrelated constructs provide more information than a smaller set of independent domains should be evaluated empirically for each system.

Fifth, ED provides additional insight into dimension-reduction techniques such as factor analysis (Fleming and Kroeske, 2013). Factor analysis identifies latent dimensions that best account for covariance among variables, excluding unique variance and error. Its aim is to denoise the data and uncover the simpler, theoretically intelligible structure underlying them (Finch, 2020). By contrast, ED addresses the complementary question of how many orthogonal dimensions are required to represent the distribution of information (variance) in a dataset, but provides no insight into the nature of these dimensions. In this sense, ED also differs from commonly used retention criteria such as Kaiser-Guttman, Velicer’s MAP, and parallel analysis. Whereas traditional retention rules focus on the shared, substantively interpretable variance and suggest a discrete factor count, ED reflects the full variance structure—including common, specific, and error variance—on a continuous scale. When computed from the original variables, ED is not directly interpretable as a factor retention criterion. However, when computed from intercorrelation matrices (*Φ*), ED quantifies a property that no other index captures—the effective number of independent dimensions represented by the extracted factors. Discrepancies between the nominal factor count and ED(Φ) can therefore inform decisions about factor retention and rotation, which remain among the most contentious issues in the field (Finch, 2020; Goretzko et al., 2021).

Finally, ED quantifies the severity of the curse of dimensionality and can help mitigate it. The curse refers to the counterintuitive geometric and probabilistic properties of high-dimensional spaces (Altman and Krzywinski, 2018). Although high-dimensionality issues are less severe in personality research than in genetics or big data, they become apparent even with a modest number of variables. If caseness is defined as scoring above one standard deviation in any normally distributed domain (T ≥ 60), 15.9% of the population would exceed this threshold on a single domain. In a multidimensional taxonomy, however, the probability of exceeding the threshold on at least one dimension increases sharply: assuming independence, it approaches 57.8% across five domains and 98.7% across 25 facets—leaving only 1.3% of individuals within the nominal normal range. These figures illustrate how, as dimensionality increases, probability mass shifts away from the multivariate center, conventional univariate cutoffs lose meaning, and the likelihood of being statistically normal quickly approaches zero (Giraud, 2021). Because these calculations assume trait independence, they are only approximate; in practice, dimensions overlap substantially, so prevalence estimation should consider effective as well as nominal dimensionality. By taking ED into account, cutoff points can be calibrated to identify extreme multivariate profiles rather than isolated univariate elevations.

In this study, we demonstrate the utility of quantifying spectral information in personality taxonomies. Using large community and clinical samples, we examine five widely recognized taxonomies of normal and pathological personality. Through ED, we evaluate informational content and redundancy within each taxonomy, across multiple alternative factor solutions, and at various hierarchical levels. We also compare the structural complexity of community and clinical samples and demonstrate how diagnostic thresholds can be adjusted based on the effective dimensionality of a taxonomy.

## 2 Materials and methods

### 2.1 Measurement instruments

The Personality Inventory for DSM-5-Short Form (PID-5-SF; Krueger et al., 2012) is a 100-item self-report measure rated on a 4-point Likert scale. It was derived from the original 220-item PID-5 and assesses the 25 pathological personality facets of the DSM-5 AMPD (American Psychiatric Association, 2013), organized into five higher-order domains: negative affect, detachment, antagonism, disinhibition, and psychoticism. The Spanish version has demonstrated good psychometric properties (Díaz-Batanero et al., 2019).

The Personality Inventory for ICD-11 (PiCD; Oltmanns and Widiger, 2018) is a 60-item self-report instrument assessing the five personality domains of the ICD-11 dimensional personality model: negative affectivity, detachment, dissociality, disinhibition, and anankastia (World Health Organization, 2018). Items are rated on a 5-point Likert scale. The Spanish version has shown adequate reliability and validity (Gutiérrez et al., 2021a).

The Dimensional Assessment of Personality Pathology – Basic Questionnaire (DAPP-BQ; Livesley and Jackson, 2009) is a 290-item self-report measure rated on a 5-point Likert scale. It was developed through factor analysis of 100 pathological personality descriptors, yielding 18 primary traits grouped into four higher-order domains: emotional dysregulation, dissocial behavior, inhibition, and compulsivity. A third hierarchical level of 72 narrower subfacets has been identified, capturing residual multidimensionality within facets (Gutiérrez et al., 2021b). The Spanish version has been validated (Gutiérrez-Zotes et al., 2008).

The Personality Diagnostic Questionnaire-4 + (PDQ-4+; Hyler, 1994) is a 99-item true/false self-report measure aligned with DSM personality disorder criteria. It assesses the 10 official DSM-5 personality disorder categories as well as negativistic and depressive personality disorders. The Spanish version has demonstrated satisfactory psychometric properties (Calvo et al., 2012).

The Temperament and Character Inventory-Revised (TCI-R; Cloninger et al., 1999) is a 140-item self-report instrument based on Cloninger’s biosocial model of personality. It assesses four temperament dimensions—novelty seeking, harm avoidance, reward dependence, and persistence—and three character dimensions—self-directedness, cooperativeness, and self-transcendence—subdivided into 29 facets. The Spanish version has shown robust psychometric properties (Gutiérrez-Zotes et al., 2015).

In addition to the personality instruments, 11 life-outcome variables were collected across all samples: academic degree, age at first employment, job category, maximum tenure in a single job, monthly income, lifetime number of romantic partners, duration of the longest romantic relationship, number of children, age at first reproduction, number of close friends, and subjective health status.

### 2.2 Sample

The community sample comprised 11,783 participants (57.6% female; age range: 14–92 years; *M* = 39.3, SD = 17.9). Of these, 5,318 completed the PID-5-SF, 5,943 the PiCD, 2,075 the DAPP-BQ, 3,141 the TCI-R, 785 the PDQ-4+, and 2,067 the life-outcome questionnaire; 29% completed at least two measures. Participants were unpaid volunteers recruited through students and their relatives or acquaintances at six Spanish universities, training centers, and non-psychiatric medical facilities.

The clinical sample comprised 5,026 outpatients (61.0% female, age range: 12–82 years; M = 36.8, SD = 12.9). Among them, 2,635 completed the PID-5-SF, 1,623 the PiCD, 2,110 the DAPP-BQ, 2,035 the TCI-R, 1,725 the PDQ-4+, and 1,044 the life-outcome questionnaire; 56% completed at least two measures. Clinical participants were recruited from mental health services at eight public and private hospitals in Spain. Diagnoses were established by experienced psychologists and psychiatrists using routine, non-structured clinical interviews conducted according to standard practice guidelines at each center. Approximately 30% of patients received a primary diagnosis of personality disorder; 26% were diagnosed with mild-to-moderate affective disorders (predominantly unipolar depression); 19% with anxiety disorders (including phobias); and 16% with mixed anxious-depressive presentations. An additional 9% presented other conditions—including eating disorders, substance-related disorders, trauma-related disorders, impulse-control disorders, and somatoform disorders—each with an individual prevalence below 5%.

Analyses were conducted using available-case (pairwise) data within each instrument or instrument combination, resulting in sample sizes that vary across analyses (see Table 1). Some cross-instrument comparisons are not fully independent; however, because ED estimates derive from instrument-specific correlation matrices rather than between-person statistics, dependency affects the precision of comparisons rather than the validity of individual instrument estimates. The study was approved by the ethics committee of the coordinating center (CEIm, Hospital Clínic de Barcelona, HCB/2025/0441).

**Table 1:** Effective dimensionality, redundancy, and information retention across hierarchical levels of personality measures.

Scales	Information		Information loss	
	ED [95% CI]	Redundancy (%)	vs. lower level (%)	vs. item level (%)
PID-5-SF (*n* = 7,953)				
5 domains	3.46 [3.41, 3.52]	30.8%	72.3%	92.1%
25 facets	12.47 [12.22, 12.69]	50.1%	71.4%	71.4%
100 items	43.58 [42.53, 44.18]	56.4%	—	—
PiCD (*n* = 7,566)				
5 domains	4.08 [4.04, 4.11]	18.4%	88.5%	88.5%
60 items	35.35 [34.82, 35.63]	41.1%	—	—
DAPP-BQ (*n* = 4,185)				
4 domains	3.38 [3.34, 3.41]	15.5%	58.9%	96.7%
18 facets	8.22 [8.03, 8.40]	54.3%	66.4%	92.1%
72 subfacets	24.49 [23.70, 24.99]	66.0%	76.4%	76.4%
290 items	103.63 [98.00, 103.1]	64.3%	—	—
PDQ-4 + (*n* = 2,510)				
3 clusters	2.25 [2.20, 2.30]	25.0%	69.7%	96.6%
12 disorders	7.43 [7.22, 7.62]	38.1%	88.8%	88.8%
92 items	66.36 [64.22, 66.17]	27.9%	—	—
TCI-R-140 (*n* = 5,176)				
7 dimensions	5.97 [5.92, 6.01]	14.7%	66.9%	92.8%
29 facets	18.04 [17.80, 18.19]	37.8%	78.2%	78.2%
140 items	82.78 [80.89, 83.74]	39.1%	—	—

ED = effective dimensionality. Redundancy (%) = percentage of nominal dimensionality that is shared owing to trait intercorrelations. Information loss (%) = percentage of reduction in ED relative to either the immediately lower level or the item-level. PID-5-SF = Personality Inventory for DSM-5—Short Form; PiCD = Personality Inventory for ICD-11; DAPP-BQ = Dimensional Assessment of Personality Pathology—Basic Questionnaire; PDQ-4 + = Personality Diagnostic Questionnaire-4+; TCI-R-140 = Temperament and Character Inventory—Revised.

### 2.3 Data analysis

Effective dimensionality was estimated using the *n*_1_ index derived from Shannon entropy, following Del Giudice (2021). The *n_1_* index distributes weight more evenly across eigenvalues than alternative estimators, reducing the dominance of larger components (Durkee et al., 2022). This property is particularly relevant in personality data, where a limited number of broad factors typically account for substantial variance while smaller components still contribute to the overall correlational structure. Compared with *n_2_* (Del Giudice, 2021, p. 533), which weights large eigenvalues more heavily and thus yields more conservative ED estimates, *n_1_* provides a broader characterization of spectral complexity and non-redundancy.

To obtain *n*_1_, the eigenvalues (*λ*) of the symmetric covariance or correlation matrix are derived via principal component analysis (PCA) and normalized by dividing each by the sum of the *k* eigenvalues. This yields a distribution of values (p_i_) that sum to 1. Although formally analogous to probabilities, these values represent a spectral normalization of variance contributions, rather than parameters of a generative probabilistic model.

Spectral entropy is computed as the sum of the *k* normalized values multiplied by the natural logarithm of each (Equation 1):


$$H(\lambda )=-\sum_{i=1}^{k}(\frac{\lambda_{j}}{\sum_{i=1}^{k}\lambda_{i}})\times \ln(\frac{\lambda_{j}}{\sum_{i=1}^{k}\lambda_{i}})=-\sum_{i=1}^{k}p_{i}\times \ln(p_{i})$$ (1)

H(λ) summarizes the dispersion of variance across orthogonal components and ranges from 0 (all variance concentrated in a single component) to ln(*k*) (variance equally distributed across components). Effective dimensionality is then obtained as *n*_1_ = *e^H^*, or equivalently (Equation 2):


$$n_{1}=\prod_{j=1}^{k}{\left(\frac{\lambda_{j}}{\sum_{i=1}^{k}\lambda_{i}}\right)}^{-(\;\frac{\lambda_{j}}{\sum_{i=1}^{k}\lambda_{i}})}=\prod_{j=1}^{k}{p_{i}}^{-p_{i}}$$ (2)

which corresponds to the effective number of orthogonal dimensions required to reproduce the observed spectral dispersion. For full mathematical foundations, see Del Giudice (2021), Cangelosi and Goriely (2007), and Gnedenko and Yelnik (2016).

Effective dimensionality was computed in R using the implementation provided by Del Giudice (2021). To ensure scale invariance and comparability across instruments and levels of aggregation, ED was applied to Pearson correlation matrices rather than covariances, thereby avoiding the confounding of structural complexity with differences in variable scale. ED depends exclusively on the relative dispersion of eigenvalues and reflects the effective number of non-redundant orthogonal variance components rather than total variance, factor loadings, or the nominal number of extracted factors. Because it is defined over the full spectral decomposition of the observed matrix, ED does not assume a latent-variable model and serves as a model-agnostic descriptive index of spectral information.

Sampling variability was evaluated via nonparametric bootstrapping with 2,000 resamples to obtain 95% confidence intervals. All analyses were conducted in R 4.3.2 (R Core Team, 2021) within RStudio (RStudio Team, 2020). Specific procedural details for each analytic context are reported in the corresponding Results sections. Data and analysis code are publicly available (Gutiérrez, 2025, 2026).

## 3 Results

### 3.1 Simulation 1: ED behavior under controlled conditions

Monte Carlo simulations established an interpretive framework for ED by systematically varying data structure and measurement properties. We generated data with 3–7 latent factors and varying inter-factor correlations (*ρ* = 0, 0.30, 0.60) and indicator reliabilities (0.60, 0.80). Each factor was measured by six indicators with primary loadings *λ* = 0.60. The base condition used continuous normally distributed data with *N* = 1,000 and no cross-loadings; extensions examined cross-loadings (λ_cross_ = 0.20), ordinal symmetric and positively skewed response formats (five-point Likert scale), and sample size variation (*N* = 200, 500, 2,000). Each condition included 1,000 replications using the MASS package (Venables and Ripley, 2002).

#### 3.1.1 Effective dimensionality versus latent dimensionality

**Figure 1:**
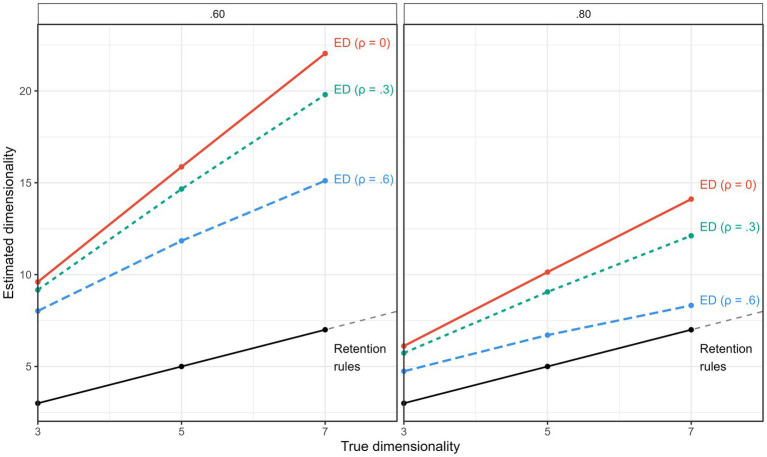
Effective dimensionality (ED) as a function of true latent dimensionality, reliability, and inter-factor correlation. Effective dimensionality (ED) is plotted against the true number of latent factors under varying levels of reliability (0.60, 0.80) and inter-factor correlation (*ρ* = 0, 0.30, 0.60). Standard retention rules (parallel analysis, MAP, and EGA) recovered the true factor count across conditions (diagonal reference black line, ED = *k*).

Standard retention rules—parallel analysis (PA; Horn, 1965), Velicer’s MAP (Velicer et al., 2000), and bootstrapped EGA (Golino and Christensen, 2024)—accurately recovered the true factor count across the primary simulation conditions. By contrast, ED increased monotonically with true factor count but consistently exceeded it (Figure 1 and Supplementary Table S1), consistent with ED quantifying spectral complexity rather than latent dimensionality. For example, under ideal conditions (reliability = 0.80, *ρ* = 0, *N* = 1,000), ED averaged 6.14, 10.2, and 14.2 for three, five, and seven true factors, respectively. This inflation was highly systematic (mean SD = 0.12, range = 0.03–0.26 across replications, representing 0.40–0.60% of the mean ED values) and was moderated by reliability and inter-factor correlation. Reducing reliability to 0.60 increased ED by 57% across all dimensionalities (*k* = 3, 5, 7), reflecting absorption of non-shared variance into the eigenvalue spectrum. Inter-factor correlation compressed ED by reducing the independence of factor-driven variance components. Sample size affected variability but not systematic bias, confirming that the relationship between ED and latent dimensionality is a structural property of the index rather than a sampling artifact. These results provide the interpretive key for the gap between nominal and effective dimensionality (see Section 3.2).

#### 3.1.2 Hierarchical aggregation

**Figure 2:**
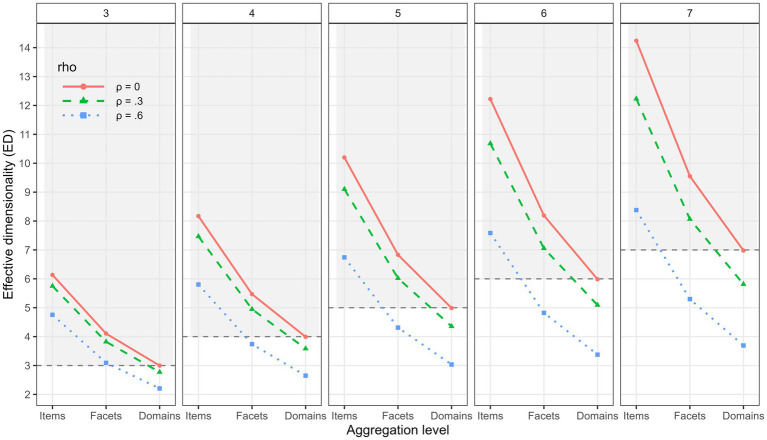
Effective dimensionality (ED) across hierarchical aggregation levels as a function of latent dimensionality and inter-factor correlation. Effective dimensionality (ED) is shown across hierarchical aggregation levels (items, facets, domains) for different levels of latent dimensionality (*k* = 3–7) and inter-factor correlation (*ρ* = 0, 0.30, 0.60).

ED decreased systematically across hierarchical levels (Figure 2 and Supplementary Table S2). Under orthogonality (*ρ* = 0) and high reliability (0.80), domain-level ED converged to the true factor count (ED = 3.00, 3.99, 4.99, 5.99, 6.98), because non-shared variance is suppressed from the spectrum. Under correlated structures (ρ = 0.60), domain-level ED fell below the true factor count, and compression increased with dimensionality: 26% for 3-factor models (ED = 2.21), 39% for 5-factor models (ED = 3.03), and 47% for 7-factor models (ED = 3.69). These results provide the basis for interpreting ED across the personality hierarchy (see Section 3.3).

#### 3.1.3 Overfactoring and redundancy

**Figure 3:**
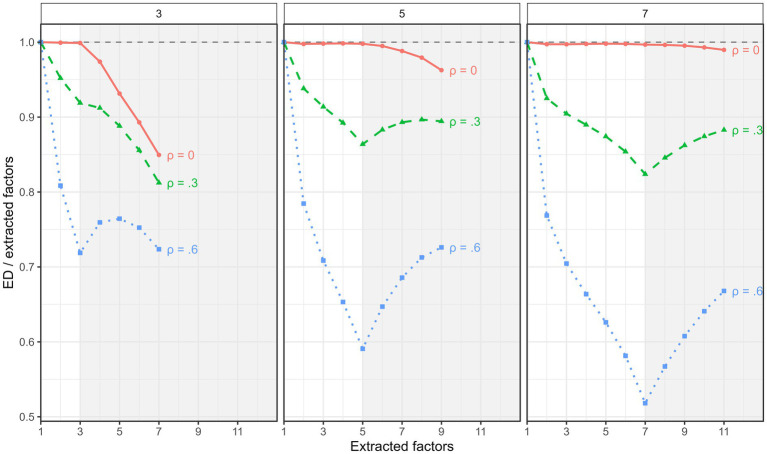
Ratio of effective to extracted dimensionality (ED/k) as a function of overfactoring and inter-factor correlation. The ratio of effective dimensionality to the number of extracted factors (ED/*k*) is plotted as a function of the number of extracted factors. Panels correspond to different number of true factors (3, 5, and 7) and lines to inter-factor correlations (*ρ* = 0, 0.30, 0.60). Values progressively below 1 indicate increasing redundancy among extracted factors; non-monotonic patterns under correlated structures reflect redistribution of variance under overfactoring.

To evaluate whether ED can detect informational redundancy in factor solutions, we extracted *k* = 1 to true factors + 4 from the simulated item-level data and computed ED from the resulting intercorrelation matrices *Φ* (Supplementary Table S3). Figure 3 displays the ratio of effective to extracted dimensionality (ED/*k*) as a function of overfactoring. Under orthogonality (ρ = 0), ED/k declined monotonically from unity, indicating that additional factors increased ED at a decreasing rate—signaling diminishing returns and emerging redundancy. Correlated structures (ρ = 0.30, 0.60) exhibited more pronounced deviations from unity, with the degree of deviation increasing with inter-factor correlation.

Notably, the relationship between extracted factors and ED/k was non-monotonic under correlated structures: initial extraction produced steep ratio declines, reached a minimum near the true dimensionality, then recovered systematically with continued overfactoring, with the recovery magnitude increasing with both inter-factor correlation and dimensionality. This pattern suggests that extreme overfactoring redistributes variance across a larger set of components, paradoxically increasing statistical independence despite the extraction of spurious factors. These results establish that ED computed from Φ detects the degree of independence and informational redundancy among extracted factors (see Section 3.4).

### 3.2 Spectral information content of personality questionnaires

After establishing ED properties through simulation, we applied it to quantify the spectral information provided by the five personality instruments: PID-5-SF, PiCD, DAPP-BQ, PDQ-4+, and TCI-R (Table 1). In all cases, ED was lower than the nominal trait count, consistent with inter-factor correlation compressing the multivariate structure. At the domain level, the TCI-R provided the most spectral information (5.97 effective dimensions), followed by the PiCD, PID-5-SF, DAPP-BQ, and PDQ-4 + (2.25 effective dimensions). At the facet level, the TCI-R again yielded the highest ED (18.04), followed by the PID-5-SF and DAPP-BQ facets, and then the 12 PDQ-4 + disorders (7.43). The DAPP-BQ was the only instrument with a third hierarchical level (72 subfacets), corresponding to 24 effective dimensions. ED estimates were highly stable across instruments and levels, with narrow 95% confidence intervals (averaging 3–4% of mean ED values) and preserved rank ordering.

Results can also be expressed as the percentage of redundant information (Table 1), calculated as (Equation 3):


$$100\times \frac{\text{nominal traits}-\mathrm{ED}}{\text{nominal traits}}$$ (3)

Redundancy quantifies the proportion of nominal dimensions that do not contribute independent variance. As a purely structural property, it does not imply inefficiency or conceptual redundancy, as correlated constructs may reflect substantive relationships or hierarchical organization (see Discussion). At the domain level, 14.7% (TCI-R) to 30.8% (PID-5-SF) of nominal dimensionality was redundant owing to trait intercorrelations. At the facet level, redundancy ranged from 37.8% (TCI-R) to 54.3% (DAPP-BQ), and reached 66.0% for DAPP-BQ subfacets.

Sensitivity analyses included ED values disattenuated using omega total (ω_t_) and ED recomputed using polychoric and tetrachoric correlation matrices for ordinal and binary items, respectively (Supplementary Table S4). Disattenuated estimates should be interpreted cautiously because reliability is strongly influenced by non-shared variance including both stochastic error and systematic item-specific variance; disattenuation may therefore overcorrect ED by removing variance that legitimately contributes to the observed spectral structure. Overall, sensitivity analyses yielded moderately lower ED estimates, with more pronounced reductions for polychoric/tetrachoric-based ED (mean *Δ* = −26.5%, range = −16 to −39% across instruments). However, the relative ordering of instruments and hierarchical levels was preserved across all specifications, supporting the robustness of comparative conclusions.

ED can also be applied jointly to multiple instruments to assess their overlap and the incremental information each provides. For example, a combined analysis of the PID-5-SF and PiCD (n = 4,243) yielded an ED of 5.69, representing a 64.5% increase relative to the PID-5-SF alone and a 39.5% increase relative to the PiCD alone (Supplementary Table S5). Combinations of the PID-5-SF or PiCD with the TCI-R yielded the highest ED estimates (7.78 and 8.08, respectively). By contrast, the DAPP-BQ added comparatively little incremental information when combined with either instrument, reflecting mutual overlap.

Finally, ED permits descriptive comparison of structural complexity across populations. Although pathological traits have been hypothesized to exhibit greater intercorrelation than normal-range traits owing to shared dysfunction (Morey et al., 2022), this was not confirmed in our data: domain overlap was lower for the TCI-R (a normal-range instrument) than for some pathological instruments (PDQ-4 + and PID-5-SF) but not others (DAPP-BQ and PiCD; Table 1). Furthermore, ED differences between community and clinical samples at the domain level were non-significant for the PID-5-SF (Δ = −0.04 [−0.14, 0.07]), PiCD (Δ = −0.08 [−0.16, 0.00]), PDQ-4 + (Δ = −0.08 [−0.19, 0.02]), and TCI-R (Δ = −0.01 [−0.11, 0.07]), whereas the DAPP-BQ showed significantly lower redundancy in the clinical sample (Δ = 0.10 [0.02, 0.16]) (Supplementary Table S6). Thus, redundancy patterns are driven more by instrument characteristics than by the pathological nature of the traits.

### 3.3 Information gain and loss across hierarchical levels

ED quantifies changes in spectral information as one moves up or down the personality hierarchy. Simulation 1 (Section 3.1.2, Figure 2) showed that ED decreases systematically during hierarchical aggregation, as averaging items into composites suppresses item-specific and error variance. In the empirical data, averaged across instruments, 82.6% of item-level spectral information was lost in moving to facets, with an additional 67.0% reduction from facets to domains (Table 1). Overall, only 6.7% of item-level spectral information was retained at the domain level. While part of this loss likely reflects random error, some may represent meaningful individual differences absent at higher levels.

To examine the functional relevance of variance lost during aggregation, we predicted 11 life outcomes from personality scores at each hierarchical level using ridge regression. Ridge regression shrinks coefficients while retaining all predictors, making it appropriate for comparisons across levels with widely varying predictor counts (3–290 across instruments and levels). Models were estimated using 10 × 10-fold cross-validation with the caret package (Kuhn, 2008). Penalty parameters (*λ*) were selected through nested cross-validation, using an inner 10-fold procedure within each training fold and a logarithmic grid spanning 10^−4^ to 10^4^. Cross-validated *R*^2^ was the primary performance index, as training *R*^2^ reflects in-sample fit and may be inflated at high dimensionality.

Predictive performance across hierarchical levels is summarized in Table 2. Item-level representations outperformed domain-level representations in cross-validated *R*^2^ across all instruments (mean difference = 0.05, range 0.04–0.07). To isolate incremental predictive information, we residualized each level against the immediately higher level and predicted outcomes from the residuals (Table 2). Residualized facet and item components yielded cross-validated *R*^2^ values ranging from 0.03 to 0.05 (means = 0.040 and 0.042, respectively), representing 67–68% of domain-level *R*^2^ across instruments (mean = 0.062), indicating substantial unique predictive variance not captured by broader dimensions.

**Table 2:** Predictive performance (cross-validated ridge regression) and prevalence of extreme scores across hierarchical levels of personality measures.

Scales		Ridge regression (*R*^2^)				% with T ≥ 70
		Raw traits		Residualized traits		
	In-sample	Cross-validated	Δ*R*^2^ vs. higher level [95% CI]	In-sample	cross- validation	
PID-5-SF (*n* = 7,953)						
5 domains	0.06	0.06	—	—	—	7.7%
25 facets	0.10	0.09	0.03 [−0.01, 0.08]	0.05	0.04	25.2%
100 items	0.16	0.11	0.02 [−0.03, 0.06]	0.07	0.03	—
PiCD (*n* = 7,566)						
5 domains	0.05	0.05	—	—	—	9.1%
60 items	0.15	0.09	0.04 [−0.02, 0.12]	0.11	0.05	—
DAPP-BQ (*n* = 4,185)						
4 domains	0.06	0.07	—	—	—	7.6%
18 facets	0.12	0.09	0.02 [−0.07, 0.12]	0.07	0.05	17.4%
72 subfacets	0.20	0.11	−0.01 [−0.12, 0.10]	0.13	0.04	43.4%
290 items	0.39	0.13	0.01 [−0.08, 0.11]	0.28	0.04	—
PDQ-4 + (*n* = 2,510)						
3 clusters	0.06	0.07	—	—	—	5.1%
12 disorders	0.09	0.08	0.01 [−0.05, 0.07]	0.03	0.03	15.9%
92 items	0.22	0.11	0.02 [−0.07, 0.12]	0.15	0.05	—
TCI-R-140 (*n* = 5,176)						
7 dimensions	0.06	0.06	—	—	—	12.1%
29 facets	0.13	0.10	0.03 [−0.04, 0.11]	0.07	0.05	34.3%
140 items	0.23	0.13	0.01 [−0.08, 0.08]	0.13	0.04	—

Δ*R*^2^ = incremental variance explained relative to the immediately higher level. Residualized traits = scores after partialling out the higher level. Percentage with *T* ≥ 70 = prevalence that this trait-level cutoff would produce if applied at the profile level. PID-5-SF = Personality Inventory for DSM-5—Short Form; PiCD = Personality Inventory for ICD-11; DAPP-BQ = Dimensional Assessment of Personality Pathology—Basic Questionnaire; PDQ-4 + = Personality Diagnostic Questionnaire-4+; TCI-R-140 = Temperament and Character Inventory—Revised.

Training *R*^2^ differences between levels were 2- to 5.5-fold larger than cross-validated differences, suggesting that lower-level models captured sample-specific noise in addition to generalizable signal. Consistent with this interpretation, ordinary least squares (OLS) regression produced larger training–validation gaps than ridge regression (Supplementary Table S7). Although item-level representations consistently outperformed domain-level representations across instruments, all 95% cross-validation resampling intervals for between-level differences included zero. Sensitivity analyses using Poisson and ordinal regression for count (*k* = 3) and ordinal (*k* = 2) outcomes produced similar patterns (Supplementary Table S7), with slightly attenuated *R*^2^ values consistent with restricted outcome variance.

Overall, these findings suggest that ED quantifies meaningful predictive information that is progressively lost through aggregation. Lower hierarchical levels retained substantial unique variance, although the resulting gains in out-of-sample prediction were modest and accompanied by greater susceptibility to overfitting.

### 3.4 ED and redundancy in factor solutions

As established in Section 3.1.3, ED and factor retention rules address different aspects of data structure and should be viewed as complementary. When computed from factor intercorrelation matrices *Φ*, ED is sensitive to the degree of covariance concentration and redundancy among extracted factors. Once a factor solution has been selected—through retention procedures or theoretical considerations—ED may quantify the effective number of independent dimensions it represents: if ED(Φ) is close to the nominal factor count *k*, factors are relatively differentiated; if substantially smaller, the solution is informationally unparsimonious.

As an application of this property, we examined how rotation method influenced ED across the five instruments. Factor solutions ranging from two to ten dimensions were extracted using minimum residual (MinRes) estimation and rotated using one orthogonal method (Simplimax) and six oblique methods (Biquartimin, oblique CF-Equamax, GeominQ, Oblimin, Promax, and BentlerQ). Analyses were performed with the R packages psych (Revelle, 2025) and GPArotation (Bernaards and Jennrich, 2005); ED was computed from the resulting Φ matrices.

Across instruments, ED/k ratios varied systematically by rotation family (Supplementary Table S8). Orthogonal and weakly oblique methods (Simplimax, Biquartimin) produced ED values close to the nominal factor count, with ED/k averaging 0.99 (SD = 0.04) and 0.94 (SD = 0.07), respectively. They are taken as a reference point, as orthogonality imposes an identity Φ matrix such that ED equals *k* by definition. Intermediate oblique methods (CF-Equamax, GeominQ, Oblimin) averaged 0.85 (SD = 0.10), and highly oblique methods (Promax, BentlerQ) averaged 0.75 (SD = 0.13), indicating greater covariance concentration. Rotation effects were highly consistent across instruments (Kendall’s concordance *W* = 0.94), whereas differences across instruments were comparatively modest.

We also examined whether ED was associated with the distribution of predictive variance across factors in a subset of theoretically supported solutions (3–5 factors for the PID-5-SF; 4–5 for the PiCD; 4 for the DAPP-BQ; 3 for the PDQ-4+; 6–7 for the TCI-R). Factor scores were used to predict the 11 life-outcome variables. Although *R*^2^ is invariant across rotations, solutions with higher ED showed predictive effects distributed across a larger number of factors (mean Spearman’s *ρ* = 0.41). For example, Simplimax solutions yielded slightly more significant predictors on average than BentlerQ solutions (2.9 vs. 2.4), with intermediate rotations falling between these extremes.

In sum, ED captures aspects of structural organization not reflected in factor count alone, by distinguishing solutions with similar factor counts in terms of how much independent versus overlapping variance they represent. Solutions with higher ED tend to distribute predictive information more broadly across factors.

### 3.5 ED and the curse of dimensionality

Effective dimensionality quantifies the severity of the curse of dimensionality in multivariate personality systems and provides a basis for mitigating its practical consequences. Whereas clinical and administrative decisions—such as personality disorder diagnoses and disability determinations—are conventionally dichotomous and should reflect overall extremity, cutoffs for caseness are typically established at the level of individual traits. When applied across multiple dimensions, such cutoffs severely inflate prevalence rates. Table 2 (right) illustrates this inflation using the conventional cutoff of *T* ≥ 70 (2.3% probability under normality). Resulting caseness ranges from 5.1 to 12.1% at the domain level (a 222–526% increase) and from 15.8 to 34.3% at the facet level (a 691–1,491% increase). Thus, the probability of being classified as extreme is driven more by dimensionality than by true trait extremity.

To maintain a fixed proportion of extreme cases across multiple dimensions, per-dimension cutoffs must be adjusted as a function of dimensionality according to (Equation 4):


c=Φ−1(p1d) (4)

where Φ^−1^ is the probit function, *p* is the desired profile-level cumulative probability, and *d* is the dimensionality parameter. We compared three approaches for specifying *d*. The nominal-dimensionality approach sets *d* = *k*, where *k* is the number of nominal traits. For example, for PID-5-SF facets (*k* = 25) and a target probability of *p* = 0.97725 (*T* > 70), this yields *c* = Φ^−1^(0.97725^1/25^) = 3.09 (*T* ≈ 81). This approach assumes trait independence and becomes overly conservative when traits are correlated. The effective-dimensionality approach sets *d* = ED, thereby accounting for trait redundancy. For PID-5-SF facets, ED = 12.47 yields *c* = 2.90 (T ≈ 79). However, ED may underestimate the dimensionality relevant for extreme-value behavior because it is derived from covariance structure averaged across the entire distribution (Huser and Wadsworth, 2022). Finally, a shrinkage approach (ED_shrunk) specifies $d=\sqrt{k\times \mathrm{ED}}$ , interpolating between nominal and effective dimensionality. For PID-5-SF facets, *d* ≈ 17.7, yielding c = 3.00 (T ≈ 80), potentially balancing the opposing biases.

### 3.6 Simulation 2: ED cutoff recalibration

We evaluated all three approaches against an unadjusted baseline (any trait ≥ 70) using Monte Carlo simulations to examine both calibration accuracy (i.e., whether methods yield the target 2.3% prevalence) and validity (i.e., whether identified cases match predefined ground truth) under realistic psychometric conditions. Data were generated with *N* = 500 and 2,000; dimensionality *k* = 5, 10, 20, 30, 50 and 70; equicorrelations *ρ* = 0, 0.20, 0.40, and 0.60; and reliabilities *α* = 1.0, 0.80, and 0.60. To assess distributional robustness, data were generated under multivariate normal and skewed distributions (skewness = 0.50 and 1.0) via a monotonic exponential transformation. Ground truth was defined via Mahalanobis distance from the population centroid with cutoffs at T = 60 (15.87% prevalence), 65 (6.68%), and 70 (2.28%). All estimates were based on 1,000 replications per condition. Monte Carlo standard errors for caseness rates ranged from 0.0002 to 0.00035, substantially smaller than the observed between-method differences. Results were consistent across sample sizes and reliability levels, and are therefore collapsed across these factors.

**Figure 4:**
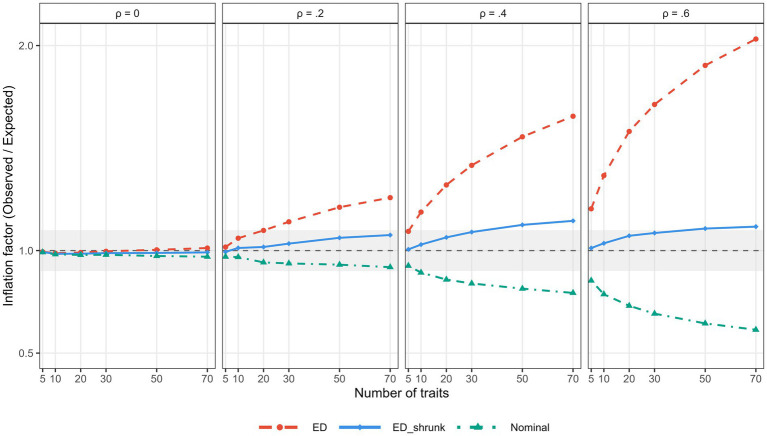
Inflation of multivariate extreme classification for nominal, ED, and ED_shrunk approaches as a function of dimensionality and intercorrelation. Inflation factor (observed-to-expected prevalence ratio) for extreme classification (*T* > 70) is plotted as a function of the number of traits (*k*) under varying levels of inter-trait correlation (*ρ* = 0, 0.20, 0.40, 0.60). Lines represent adjustment methods: nominal (independence-based adjustment), ED (multivariate aggregation), and ED_shrunk (shrinkage-adjusted effective dimensionality). The dashed horizontal line indicates perfect calibration (inflation = 1). The shaded band represents a heuristic acceptable deviation range (±10%).

Calibration performance under multivariate normality at T > 70 is summarized in Figure 4, which displays the inflation factor (observed-to-expected prevalence ratio) as a function of dimensionality and intercorrelation. Under independence (*ρ* = 0), nominal and effective dimensionality converge by definition, and all methods produced well-calibrated estimates (inflation ≈ 1.0 across *k* = 5–70). In contrast, the unadjusted ‘any trait’ rule (not shown in Figure 4) produced severe inflation even under independence, ranging from 4.8× at *k* = 5 to 35.1× at *k* = 70 (Supplementary Table S9).

As intercorrelation increased, adjusted methods diverged systematically. Nominal adjustment became progressively conservative, underestimating prevalence by 39% at *ρ* = 0.60 and *k* = 70 (caseness rate = 1.40%, inflation 0.61), whereas the ED adjustment overestimated it by 103% (caseness rate = 4.63%, inflation = 2.03). This divergence is expected from an extreme-value theory perspective, as nominal dimensionality assumes tail independence, while ED is derived from dependence structure in the distribution bulk and may not fully capture dependence in the tails (Huser and Wadsworth, 2022). By contrast, the ED_shrunk estimator yielded an intermediate value (caseness rate = 2.55%, inflation = 1.12), balancing the opposing biases and maintaining inflation within approximately 1–15% of the target across ρ = 0.20–0.60 and *k* = 20–70.

All methods deteriorated markedly under skewed distributions (Supplementary Table S9), although their relative ordering remained stable. For the adjusted approaches, inflation typically ranged 2- to 6-fold at lower dimensionality (*k* ≤ 20) and 5- to 14-fold at higher dimensionality (*k* ≥ 50). The unadjusted ‘any trait’ rule showed far more extreme inflation, ranging from approximately 3.5× to over 37.0× across conditions. Thus, dimensionality adjustments depend critically on multivariate normality and offer limited protection against distributional misspecification.

**Figure 5:**
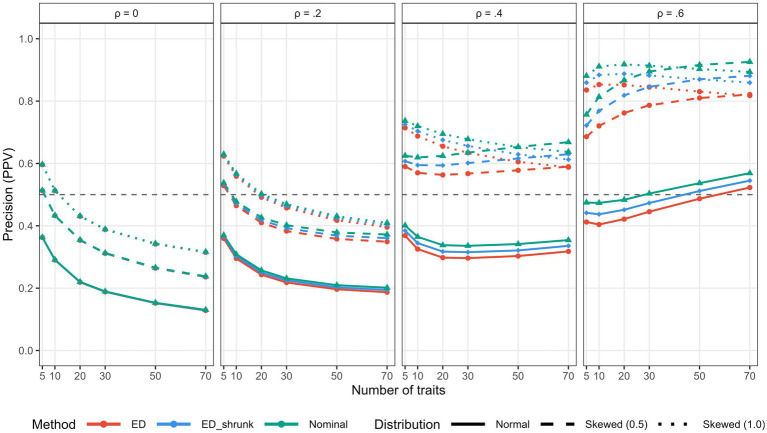
Precision (positive predictive value) of dimensionality-adjusted cutoff methods as a function of trait dimensionality, intercorrelation, and distributional form. Precision (PPV) is plotted against the number of traits (*k* = 5–70) for three adjustment methods (Nominal, ED, ED_shrunk) under varying intercorrelations (*ρ* = 0, 0.20, 0.40, 0.60) and distributions (Normal, skewed 0.5, skewed 1.0). The horizontal dashed line indicates the base rate (prevalence = 2.28% at *T* = 70).

Beyond calibration, validity analyses revealed context-dependent performance patterns. Overall agreement with Mahalanobis distance (Jaccard index; Supplementary Figure S1) declined substantially as dimensionality and intercorrelation increased, reflecting fundamental differences between threshold-based and distance-based definitions of multivariate extremity. Sensitivity showed similar declines, falling below 0.10 for all methods at *k* = 70 and ρ = 0.60 under normality (Supplementary Figure S2). Precision (positive predictive value) revealed a more complex pattern that varied with correlation structure (Figure 5). Under independence (ρ = 0, 0.20), precision declined with dimensionality across all distributions. This pattern reversed at moderate to high intercorrelation (ρ ≥ 0.40), where precision stabilized or modestly increased with dimensionality, particularly under skewed distributions. At ρ = 0.60 and skewness = 1.0, precision reached 0.80–0.92 at *k* = 70, indicating that threshold-based methods increasingly identify true multivariate extremes when traits are substantially intercorrelated and non-normally distributed—conditions common in psychopathology research. This pattern was consistent across adjustment methods, with ED_shrunk maintaining intermediate performance.

These findings show that ED-based threshold rules improve prevalence calibration but only partially capture multivariate extremes, and are strongly governed by data structure. Whereas trait independence disperses extremity across dimensions, correlation and skewness concentrate it. As a result, threshold-based classifications diverge from distance-based definitions, identifying only a restricted subset of multivariate extremes.

## 4 Discussion

One crucial attribute of any personality system is the amount of information encoded in its variance structure. By analyzing ED in simulation data and five well-established personality systems, we have demonstrated the value of quantifying spectral information within single instruments and their combinations, as well as across hierarchical levels, alternative factor structures, and populations.

### 4.1 ED, information, and redundancy

Personality systems generally convey less spectral information than their nominal dimensionality suggests. The discrepancy is modest for the DAPP-BQ, PiCD, and TCI-R, but larger for the PDQ-4+ and the five PID-5-SF domains, which correspond to 2.3 and 3.5 effective dimensions, respectively. Thus, whereas the PID-5 has shown excellent replicability (García et al., 2024), its primary limitation may be one of parsimony. Notably, this pattern is not unique to pathological systems, as the Big Five has been found to capture slightly more than four effective dimensions on average (Del Giudice, 2021). This problem has generally been conceptualized as one of redundancy—the inverse of information. Although the DSM categorical system (here represented by the PDQ-4+) has been superseded in part because of overlap among ostensibly distinct categories, the PID-5-SF—intended as its replacement—may exhibit comparable or greater redundancy. This finding does not diminish the many advantages of dimensional approaches (Monaghan and Bizumic, 2023), but highlights that spectral information and redundancy are rarely considered explicitly and that doing so may inform instrument design and taxonomy refinement.

### 4.2 ED and orthogonality

One straightforward way to increase information in personality systems is to favor less correlated constructs (Lahey, 2023). A non-overlapping taxonomy provides a more parsimonious coordinate system for locating individuals, improves discriminant validity, and minimizes multicollinearity in predictive models. In our data, redundancy was modest for some instruments—such as the DAPP-BQ and the TCI-R— suggesting that relatively independent dimensions are empirically attainable. This demonstrates that quasi-orthogonal dimensions of negative emotionality, detachment, antagonism, and disinhibition–anankastia are not only theoretically desirable but also empirically achievable.

That said, ED does not assume trait independence—in fact, it makes no assumptions about underlying structure—and orthogonality also has its own drawbacks. First, it is plausible at the domain level but less so at the facet or lower levels, both because lower-order traits are designed to capture multiple facets of the same superordinate construct and because identifying four independent dimensions is easier than identifying thirty. Second, orthogonality may impose mathematical simplicity where none is warranted (Cattell and Tsujioka, 1964). For example, the behavioral systems assumed to underlie personality domains are not supposed to be truly independent but to interact (DeYoung, 2015). In biological and neurobehavioral systems, overlap may reflect degeneracy—the capacity of structurally distinct components to produce similar functional outcomes—which is a key source of robustness and evolvability (Whitacre, 2010). From this perspective, partially overlapping dimensions may support flexible, many-to-one or many-to-many mappings between underlying processes and observed traits, rather than constituting inefficiency. Recent theoretical and empirical work suggests that such overlaps can emerge from evolutionary trajectories shaping brain–behavior relationships and the distribution of psychological phenotypes (Di Plinio and Ebisch, 2020; Di Plinio et al., 2024). Finally, orthogonality is inconsistent with the nested, multilayered architectures of part-whole relationships that define the personality hierarchy (Findlay and Thagard, 2012). Such hierarchies are ubiquitous in nature and may reflect universal principles governing the structure and dynamics of living systems, from cells and organisms to social networks and entire societies (Goekoop and de Kleijn, 2023). In this context, independent dimensions may be a convenient fiction—much like the vectors used to decompose a ballistic trajectory.

None of this diminishes—rather, it reinforces—the importance of quantifying the information encoded in personality taxonomies. Because oblique solutions imply shared variance, it is critical to quantify how much redundancy a given solution introduces and whether increases in nominal dimensionality reflect genuinely independent information. In this respect, ED provides insights that conventional factor-retention rules miss: whereas traditional criteria suggest a statistically defensible number of factors, ED evaluates the informational parsimony of each solution and can identify over-dimensioned structures in which additional factors merely redistribute shared variance across correlated factors. Although redundancy is common in personality data, it is often overlooked in taxonomic research. Importantly, however, redundancy itself is theoretically ambiguous in that it may arise from conceptual overlap, degeneracy, hierarchical organization, shared method variance, or other sources of covariance. Its interpretation therefore depends on the substantive theoretical framework in which the taxonomy is embedded.

### 4.3 ED and hierarchical levels

A second route to increasing information is to move down the hierarchy. Individuals who appear similar at the domain level can differ substantially at lower levels of aggregation (Mõttus, 2016; Mõttus et al., 2019; Revelle, 2024). Our results reveal that items contain 9–31-fold more spectral information than domains across instruments, with a portion of this variance representing unique—not error—variance. This holds even after removing higher-level variance, in line with recent work obtaining similar effect sizes (Nielsen and Kajonius, 2024; Seeboth and Mõttus, 2018; Stewart et al., 2022).

However, increased granularity is not without cost (Cooper, 2019). As information increases, so does redundancy—rising from 21% at the domain level to 45% at the facet and item levels. This reduces informational parsimony, weakens discriminant validity, and increases collinearity in predictive models. Moreover, lower-level representations are more sensitive to estimation noise and overfitting, resulting in training–validation discrepancies and cross-validated differences that do not reach statistical significance despite non-trivial effect sizes. Finally, a lower-level taxonomy may impose substantial interpretive and practical burdens. In information-theoretic terms (Shannon, 1948), greater informativeness entails higher entropy and disorder, which does not necessarily yield greater practical utility. Personality hierarchies thus reflect a structural trade-off: lower levels are informationally richer and may enhance prediction, whereas higher levels provide ordered compression and enable interpretation.

### 4.4 ED and diagnostic thresholds

Our findings highlight the statistical improbability of remaining normal across dozens or hundreds of dimensions—a point illustrated by lifetime prevalence estimates of mental disorders approaching 83% (Schaefer et al., 2017). Such figures are more plausible than they initially appear, as the lifetime prevalence of infectious or musculoskeletal disorders is even higher. Nevertheless, a taxonomy with sufficient variables will inevitably classify most individuals as disordered, at which point it ceases to be useful. This underscores the need to distinguish individuals with isolated trait elevations—virtually everyone—from those with excessive, pervasive, and impairing configurations of traits, who are comparatively rare. High dimensionality therefore calls for a more realistic formulation of caseness (Dalgleish et al., 2020).

Although statistical extremity cannot define disorder on its own, additional required criteria—such as functioning, impairment, and need for care—are themselves multidimensional and partially overlap with one another and with personality traits. As a result, the logic of multivariate extremity applies to diagnostic decisions broadly, making dimensionality and dependence structure a general constraint irrespective of disorder definition. While not a complete solution, shrinkage between nominal and effective dimensionality provides a more stable approximation to extreme-case prevalence in high-dimensional correlated data and addresses the specific problem of threshold calibration under multivariate structures. In contrast, the observed divergence from distance-based definitions reflects differences in how extremity is operationalized and warrants further investigation.

At minimum, the focus on single traits and disorders should be complemented by metrics that characterize an individual’s position within the broader multivariate personality space. This view aligns with evidence suggesting that lifetime psychopathology and burden are better captured by the number and diversity of disorders than by any specific diagnosis (Caspi et al., 2024; Gutiérrez et al., 2023; Soeteman et al., 2008). Beyond ED, relevant approaches for quantifying relationships in multivariate spaces include kernel-based methods, Manhattan and fractional distances, cosine similarity, t-distributed stochastic neighbor embedding, and Mahalanobis distance D, among others (Chakraborty and Zhang, 2021; Del Giudice, 2013; Pandove et al., 2018). For example, Mahalanobis D quantifies the multivariate distance between an individual’s full profile and the population centroid, accounting for covariance structure. It has been shown to outperform individual trait scores, total scores, extreme-trait counts, and the general factor of personality disorder in predicting health-service utilization (Gutiérrez et al., 2023). As dimensional taxonomies expand, clinical decision-making will increasingly depend on tools capable of navigating high-dimensional personality spaces in a statistically principled manner.

### 4.5 Psychometric applications of ED

Our results show that ED can provide practical guidance across key stages of instrument development and model validation. ED quantifies how much independent variance an item pool captures relative to its nominal dimensionality, helping identify redundant items beyond traditional reliability indices. Computed from *Φ*, ED complements retention rules by revealing how much independent information a factor solution carries, and motivates revision when the result is theoretically unexpected. ED also informs aggregation decisions by quantifying how much spectral information is lost when items are combined into facets or domains, detecting trade-offs between parsimony and structural complexity that are not reflected by nominal dimensionality. When selecting instruments or test batteries, ED indexes both the spectral informativeness of each measure and its incremental contribution in combination with others, enabling principled selection rather than reliance on face validity alone. Finally, cutoffs based on shrinkage ED reduce prevalence inflation that arises mechanically from multiplying traits or disorders, supporting well-calibrated diagnostic decision-making.

### 4.6 Limitations

Several limitations should be noted. First, ED is sensitive to assessment format. ED estimates for DSM personality disorders differ substantially between questionnaire- and interview-based assessments (3.04 vs. 5.25; data from Müller et al. (2026), Supplementary Figure S2). Accordingly, findings based on self-report data should be generalized to other formats with caution. Second, predictive analyses were limited to a relatively narrow set of life outcomes available across samples, and the generalizability of predictive findings remains to be established. Third, the choice of correlation matrix—Pearson versus polychoric or tetrachoric—had a non-trivial impact on ED estimates (mean *Δ* = −26.5%), underscoring that ED quantification is not method-agnostic and that reporting choices must be made explicit. Fourth, ED is not the only framework for quantifying information, redundancy, or structural complexity. Alternative indices—including quadratic and permutation entropy, fractal dimensionality, Rényi information, and Chernoff divergence—have been applied in other disciplines depending on data properties and inferential goals (Budescu and Budescu, 2012; Crooks, 2024; Verdú, 2019). ED should therefore be viewed as one principled, covariance-based index within a broader family of information-theoretic approaches, and comparative studies are needed to delineate their relative sensitivity, interpretability, and domains of applicability.

## 5 Conclusion

Personality and psychopathology classifications are moving toward high-dimensional, multilayered taxonomies composed of partially redundant constructs (Dalgleish et al., 2020; Eaton et al., 2023). Because personality—and biological systems more broadly—is not inherently orthogonal, such taxonomies generate complex covariance architectures that require appropriate metrics to quantify how much effective information they encode. At the same time, taxonomies must remain structurally parsimonious, empirically replicable, theoretically coherent, and practically manageable if they are to function as useful maps of the psychological space (Finch, 2020).

These objectives need not be mutually exclusive. Effective dimensionality quantifies the informational value of any covariance matrix, providing a compact index that distinguishes nominal from effective dimensionality for single instruments and their combinations, compares information across hierarchical levels and populations, complements traditional factor-retention criteria by flagging unparsimonious solutions, and supports profile-level decisions by calibrating cutoffs under high-dimensionality conditions.

With established applications across disciplines—from physics and evolutionary biology to political science—Shannon entropy supplies a universal, model-agnostic, and theoretically grounded framework for addressing fundamental problems of redundancy and dimensionality. As dimensional taxonomies expand, incorporating explicit measures of informational efficiency such as ED will be essential for guiding the construction and refinement of personality measurement and models.

## Data Availability

The datasets presented in this study can be found in online repositories. The names of the repository/repositories and accession number(s) can be found in the article/Supplementary material. The data and R code files can be downloaded at https://osf.io/ky4vj/overview.

## References

[ref1] Altman N. Krzywinski M. (2018). The curse(s) of dimensionality. Nat. Methods 15, 399–400. doi: 10.1038/s41592-018-0019-x, 29855577

[ref2] American Psychiatric Association (2013). Diagnostic and Statistical Manual of Mental Disorders. 5th Edn Washington, DC: American Psychiatric Association.

[ref3] Bernaards C. A. Jennrich R. I. (2005). Gradient projection algorithms and software for arbitrary rotation criteria in factor analysis. Educ. Psychol. Meas. 65, 676–696. doi: 10.1177/0013164404272507

[ref4] Budescu D. V. Budescu M. (2012). How to measure diversity when you must. Psychol. Methods 17, 215–227. doi: 10.1037/a0027129, 22309955

[ref5] Calvo N. Gutiérrez F. Andión O. Caseras X. Torrubia R. Casas M. (2012). Psychometric properties of the Spanish version of the self-report personality diagnostic Questionnaire-4+ (PDQ-4+) in psychiatric outpatients. Psicothema 24, 156–160. 22269379

[ref6] Cangelosi R. Goriely A. (2007). Component retention in principal component analysis with application to cDNA microarray data. Biol. Direct 2:2. doi: 10.1186/1745-6150-2-2, 17229320 PMC1797006

[ref7] Caspi A. Houts R. M. Fisher H. L. Danese A. Moffitt T. E. (2024). The general factor of psychopathology (p): choosing among competing models and interpreting p. Clin. Psychol. Sci. 12, 53–82. doi: 10.1177/21677026221147872, 38236494 PMC10794018

[ref8] Cattell R. B. Tsujioka B. (1964). The importance of factor-trueness and validity, versus homogeneity and orthogonality, in test scales. Educ. Psychol. Meas. 24, 3–30. doi: 10.1177/001316446402400101

[ref9] Chakraborty S. Zhang X. (2021). A new framework for distance and kernel-based metrics in high dimensions. Electron. J. Stat. 15, 5455–5522. doi: 10.1214/21-EJS1889

[ref10] Cloninger C. R. Przybeck T. R. Svrakic D. M. (1999). The Temperament and Character Inventory-Revised. St. Louis, MO: Washington University.

[ref11] Condon D. M. Mõttus R. (2021). “A role for information theory in personality modeling, assessment, and judgment,” in Measuring and Modeling Persons and Situations, eds. Wood D. Read S. J. Harms P. D. Slaughter A. (London, UK: Elsevier Academic Press), 1–31.

[ref12] Cooper C. (2019). Pitfalls of personality theory. Pers. Individ. Dif. 151:109551. doi: 10.1016/j.paid.2019.109551

[ref13] Cover T. M. Thomas J. A. (2006). Elements of Information Theory. Hoboken, NJ: John Wiley & Sons.

[ref14] Crooks G. E. (2024) On Measures of entropy and Information [Technical note]. ThreePlusOne. Available online at: https://threeplusone.com/pubs/on_information.pdf (Accessed July 22, 2026).

[ref15] Dalgleish T. Black M. Johnston D. Bevan A. (2020). Transdiagnostic approaches to mental health problems: current status and future directions. J. Consult. Clin. Psychol. 88, 179–195. doi: 10.1037/ccp0000482, 32068421 PMC7027356

[ref16] Del Giudice M. (2013). Multivariate misgivings: is D a valid measure of group and sex differences? Evol. Psychol. 11, 1067–1076. doi: 10.1177/147470491301100511, 24333840 PMC10434404

[ref17] Del Giudice M. (2021). Effective dimensionality: a tutorial. Multivar. Behav. Res. 56, 527–542. doi: 10.1080/00273171.2020.1743631, 32223436

[ref18] DeYoung C. G. (2015). Cybernetic big five theory. J. Res. Pers. 56, 33–58. doi: 10.1016/j.jrp.2014.07.004

[ref19] Di Plinio S. Ebisch S. J. H. (2020). Combining local and global evolutionary trajectories of brain-behaviour relationships through game theory. Eur. J. Neurosci. 52, 4198–4213. doi: 10.1111/ejn.14883, 32594640

[ref20] Di Plinio S. Northoff G. Ebisch S. (2024). The degenerate coding of psychometric profiles through functional connectivity archetypes. Front. Hum. Neurosci. 18:1455776. doi: 10.3389/fnhum.2024.1455776, 39318702 PMC11419991

[ref21] Díaz-Batanero C. Ramírez-López J. Domínguez-Salas S. Fernández-Calderón F. Lozano Ó. M. (2019). Personality inventory for DSM-5–short form (PID-5-SF): reliability, factorial structure, and relationship with functional impairment in dual diagnosis patients. Assessment 26, 853–866. doi: 10.1177/1073191117739980, 29117705

[ref22] Durkee P. K. Lukaszewski A. W. von Rueden C. R. Gurven M. D. Buss D. M. Tucker-Drob E. M. (2022). Niche diversity predicts personality structure across 115 nations. Psychol. Sci. 33, 285–298. doi: 10.1177/09567976211031571, 35044268 PMC13171031

[ref23] Eaton N. R. Bringmann L. F. Elmer T. Fried E. I. Forbes M. K. Greene A. L. . (2023). A review of approaches and models in psychopathology conceptualization research. Nat. Rev. Psychol. 2, 622–636. doi: 10.1038/s44159-023-00218-4

[ref24] Finch W. (2020). Exploratory Factor Analysis. Thousand Oaks, CA: SAGE Publications, Inc.

[ref25] Findlay S. D. Thagard P. (2012). How parts make up wholes. Front. Physiol. 3:455. doi: 10.3389/fphys.2012.00455, 23227011 PMC3510642

[ref26] Fleming B. Kroeske J. (2013). An information-theoretic approach to dimension reduction of financial data. SSRN Electron. J. doi: 10.2139/ssrn.2260410

[ref27] García L. F. Gutiérrez F. García O. Aluja A. (2024). The alternative model of personality disorders: assessment, convergent and discriminant validity, and a look to the future. Annu. Rev. Clin. Psychol. 20, 431–455. doi: 10.1146/annurev-clinpsy-081122-010709, 38211624

[ref28] Giraud C. (2021). Introduction to High-Dimensional Statistics. 2nd Edn New York: Chapman & Hall.

[ref29] Gnedenko B. Yelnik I. (2016). Minimum entropy as a measure of effective dimensionality. SSRN Electron. J. doi: 10.2139/ssrn.2767549

[ref30] Goekoop R. de Kleijn R. (2023). Hierarchical network structure as the source of hierarchical dynamics (power-law frequency spectra) in living and non-living systems: how state-trait continua (body plans, personalities) emerge from first principles in biophysics. Neurosci. Biobehav. Rev. 154:105402. doi: 10.1016/j.neubiorev.2023.105402, 37741517

[ref31] Golino H. Christensen A. P. (2024). EGAnet: exploratory graph analysis – a framework for estimating the number of dimensions in multivariate data using network psychometrics. CRAN. doi: 10.32614/CRAN.package.EGAnet

[ref32] Goretzko D. Pham T. T. H. Bühner M. (2021). Exploratory factor analysis: current use, methodological developments, and recommendations for good practice. Curr. Psychol. 40, 3510–3521. doi: 10.1007/s12144-019-00300-2

[ref33] Gutiérrez F. (2025) The Effective Dimensionality of Personality Pathology [Data set]. Open Science Framework. Available online at: https://osf.io/ky4vj/files/hfsdk. (Accessed July 22, 2026).

[ref34] Gutiérrez F. (2026). The Effective Dimensionality of Personality Pathology.R [Computer code]. Open Science Framework. Available online at: https://osf.io/ky4vj/files/y7kcu (Accessed July 22, 2026).

[ref35] Gutiérrez F. Aluja A. Ruiz J. García L. F. Gárriz M. Gutiérrez-Zotes A. . (2021a). Personality disorders in the ICD-11: Spanish validation of the PiCD and the SASPD in a mixed community and clinical sample. Assessment 28, 759–772. doi: 10.1177/1073191120936357, 32583685 PMC7961637

[ref36] Gutiérrez F. Peri J. M. Aluja A. Baillés E. Sureda B. Gutiérrez-Zotes A. . (2023). Differentiating abnormal, normal, and ideal personality profiles in multidimensional spaces. J. Individ. Differ. 44, 215–222. doi: 10.1027/1614-0001/a000395

[ref37] Gutiérrez F. Vicente E. Aluja A. Peri J. M. Gutiérrez-Zotes A. Baillés E. . (2021b). A third hierarchical level of narrower traits for the dimensional assessment of personality pathology-basic questionnaire. Pers. Ment. Health 15, 239–251. doi: 10.1002/pmh.1511, 33871181

[ref38] Gutiérrez-Zotes J. A. Gutiérrez F. Valero J. Gallego E. Baillés E. Torres X. . (2008). Structure of personality pathology in normal and clinical samples: Spanish validation of the DAPP-BQ. J. Personal. Disord. 22, 389–404. doi: 10.1521/pedi.2008.22.4.389, 18684051

[ref39] Gutiérrez-Zotes A. Labad J. Martorell L. Gaviria A. Bayón C. Vilella E. . (2015). The revised temperament and character inventory: normative data by sex and age from a Spanish normal randomized sample. PeerJ 3:e1481. doi: 10.7717/peerj.1481, 26713237 PMC4690388

[ref40] Horn J. L. (1965). A rationale and test for the number of factors in factor analysis. Psychometrika 30, 179–185. doi: 10.1007/BF02289447, 14306381

[ref42] Huser R. Wadsworth J. L. (2022). Advances in statistical modeling of spatial extremes. WIREs Comput. Stat. 14:e1537. doi: 10.1002/wics.1537

[ref43] Hyler S. E. (1994). PDQ-4+ Personality Diagnostic Questionnaire-4+. New York, NY: New York State Psychiatric Institute.

[ref44] Kosman E. Feijen F. Jokela J. (2024). Effective number of different populations: a new concept and how to use it. Ecol. Evol. 14:e70303. doi: 10.1002/ece3.70303, 39279796 PMC11402519

[ref45] Krueger R. F. Derringer J. Markon K. E. Watson D. Skodol A. E. (2012). Initial construction of a maladaptive personality trait model and inventory for DSM-5. Psychol. Med. 42, 1879–1890. doi: 10.1017/S0033291711003145, 22153017 PMC3413381

[ref46] Kuhn M. (2008). Building predictive models in R using the caret package. J. Stat. Softw. 28, 1–26. doi: 10.18637/jss.v028.i0527774042

[ref47] Laakso M. Taagepera R. (1979). “Effective” number of parties: a measure with application to West Europe. Comp. Polit. Stud. 12, 3–27. doi: 10.1177/001041407901200101

[ref48] Lahey B. B. (2023). Using dispositions to understand otherwise intractable causal pathways to psychological problems during childhood and adolescence. J. Clin. Child Adolesc. Psychol. 53, 328–341. doi: 10.1080/15374416.2023.2292050, 38109688 PMC11472698

[ref49] Livesley W. J. Jackson D. (2009). Manual for the Dimensional Assessment of Personality Pathology—Basic Questionnaire. Port Huron, MI: Sigma.

[ref50] Monaghan C. Bizumic B. (2023). Dimensional models of personality disorders: challenges and opportunities. Front. Psych. 14:1098452. doi: 10.3389/fpsyt.2023.1098452, 36960458 PMC10028270

[ref51] Morey L. C. Good E. W. Hopwood C. J. (2022). Global personality dysfunction and the relationship of pathological and normal trait domains in the DSM-5 alternative model for personality disorders. J. Pers. 90, 34–46. doi: 10.1111/jopy.12560, 32422689

[ref52] Mõttus R. (2016). Towards more rigorous personality trait–outcome research. Eur. J. Personal. 30, 292–303. doi: 10.1002/per.2041

[ref53] Mõttus R. Sinick J. Terracciano A. Hřebíčková M. Kandler C. Ando J. . (2019). Personality characteristics below facets: a replication and meta-analysis of cross-rater agreement, rank-order stability, heritability, and utility of personality nuances. J. Pers. Soc. Psychol. 117, e35–e50. doi: 10.1037/pspp0000202, 30047763 PMC6348042

[ref54] Müller S. Schroeders U. Bachrach N. Benecke C. Cuevas L. Doering S. . (2026). Revisiting the structure of DSM-5 section II personality disorder criteria using individual participant data meta-analysis. Pers. Disord. 17, 1–16. doi: 10.1037/per0000736 40705663

[ref55] Nielsen M. D. Kajonius P. (2024). Beyond the big five factors: using facets and nuances for enhanced prediction in life outcomes. Curr. Psychol. 43, 18621–18630. doi: 10.1007/s12144-024-05662-w

[ref56] Oltmanns J. R. Widiger T. A. (2018). A self-report measure for the ICD-11 dimensional trait model proposal: the personality inventory for ICD-11. Psychol. Assess. 30, 154–169. doi: 10.1037/pas0000459, 28230410 PMC5930359

[ref57] Pandove D. Goel S. Rani R. (2018). Systematic review of clustering high-dimensional and large datasets. ACM Trans. Knowl. Discov. Data 12, 1–68. doi: 10.1145/3132088

[ref58] Pirkl R. J. Remley K. A. Patane C. S. L. (2012). Reverberation chamber measurement correlation. IEEE Trans. Electromagn. Compat. 54, 533–545. doi: 10.1109/TEMC.2011.2166964

[ref59] Preskill J. (2018). Quantum Shannon Theory. (Lecture notes for Physics 219/Computer Science 219). California Institute of Technology. doi: 10.48550/arXiv.1604.07450

[ref60] R Core Team (2021). R: A Language and Environment for Statistical Computing. R Foundation for Statistical Computing.

[ref61] Revelle W. (2024). The seductive beauty of latent variable models: or why I don’t believe in the easter bunny. Pers. Individ. Dif. 221, 112552. doi: 10.1016/j.paid.2024.112552, 38826717

[ref62] Revelle W. (2025). psych: Procedures for Psychological, Psychometric, and Personality Research (R package). Evanston, IL: Northwestern University. Available online at: https://CRAN.R-project.org/package=psych (Accessed July 22, 2026).

[ref63] Roy O. Vetterli M. (2007) The effective rank: a measure of effective dimensionality. In Proc. 15th Eur. Signal Process. Conf., (Poznan, Poland: EURASIP), 606–610.

[ref64] RStudio Team (2020). RStudio: Integrated Development for R RStudio, PBC. Available online at: https://www.rstudio.com/ (Accessed July 22, 2026).

[ref65] Schaefer J. D. Caspi A. Belsky D. W. Harrington H. Houts R. Horwood L. J. . (2017). Enduring mental health: prevalence and prediction. J. Abnorm. Psychol. 126, 212–224. doi: 10.1037/abn0000232, 27929304 PMC5304549

[ref66] Seeboth A. Mõttus R. (2018). Successful explanations start with accurate descriptions: questionnaire items as personality markers for more accurate predictions. Eur. J. Personal. 32, 186–201. doi: 10.1002/per.2147

[ref67] Shannon C. E. (1948). A mathematical theory of communication. Bell Syst. Tech. J. 27, 379–423. doi: 10.1002/j.1538-7305.1948.tb01338.x

[ref68] Sherwin W. B. Chao A. Jost L. Smouse P. E. (2017). Information theory broadens the spectrum of molecular ecology and evolution. Trends Ecol. Evol. 32, 948–963. doi: 10.1016/j.tree.2017.09.012, 29126564

[ref69] Soeteman D. I. Verheul R. Busschbach J. J. (2008). The burden of disease in personality disorders: diagnosis-specific quality of life. J. Personal. Disord. 22, 259–268. doi: 10.1521/pedi.2008.22.3.259, 18540798

[ref70] Stewart R. D. Mõttus R. Seeboth A. Soto C. J. Johnson W. (2022). The finer details? The predictability of life outcomes from big five domains, facets, and nuances. J. Pers. 90, 167–182. doi: 10.1111/jopy.12660, 34236710

[ref71] Stone J. V. (2015). Information Theory: A Tutorial Introduction. Sheffield, UK: Sebtel Press.

[ref72] Velicer W. F. Eaton C. A. Fava J. L. (2000). “Construct explication through factor or component analysis: a review and evaluation of alternative procedures for determining the number of factors or components,” in Problems and Solutions in Human Assessment: Honoring Douglas N. Jackson at Seventy, eds. Goffin R. D. Helmes E. (New York, NY: Kluwer Academic/Plenum Publishers), 41–71.

[ref73] Venables W. N. Ripley B. D. (2002). Modern Applied Statistics with S. 4th Edn. New York, NY: Springer.

[ref74] Verdú S. (2019). Empirical estimation of information measures: a literature guide. Entropy 21:720. doi: 10.3390/e21080720, 33267434 PMC7515235

[ref75] Whitacre J. M. (2010). Degeneracy: a link between evolvability, robustness and complexity in biological systems. Theor. Biol. Med. Model. 7:6. doi: 10.1186/1742-4682-7-6, 20167097 PMC2830971

[ref76] World Health Organization. (2018). International Statistical Classification of Diseases for Mortality and Morbidity Statistics, 11th Revision. Geneva: World Health Organization. Available online at: https://www.who.int/standards/classifications/classification-of-diseases (Accessed January 27, 2026).

