# Supplementary material for: Quantifying spectral information and redundancy in high-dimensional personality taxonomies: an entropy-based approach
Source: Front Psychol. 2026 Jul 29;17:1820233. doi: 10.3389/fpsyg.2026.1820233 (PMC13461502; doi:10.3389/fpsyg.2026.1820233)
Supplement: Supplementary file 1 [file Data_Sheet_1.pdf]

Supplementary Material: Quantifying Spectral Information and Redundancy in High-Dimensional Personality Taxonomies: An Entropy-Based Approach

Supplementary Table S1. *Simulation 1: Estimated dimensionality across methods under primary conditions (continuous indicators, no cross-loadings, N = 1000)*

	method	n_factors	rho	reliability	mean_est	sd_est	bias	rmse
1	ED	3	0	0.6	9.65	0.0811	6.65	6.65
2	ED	3	0	0.8	6.14	0.0374	3.14	3.14
3	ED	3	0.3	0.6	9.22	0.102	6.22	6.22
4	ED	3	0.3	0.8	5.75	0.0628	2.75	2.75
5	ED	3	0.6	0.6	8.06	0.119	5.06	5.06
6	ED	3	0.6	0.8	4.76	0.0722	1.76	1.76
7	ED	4	0	0.6	12.8	0.0909	8.83	8.83
8	ED	4	0	0.8	8.17	0.0436	4.17	4.17
9	ED	4	0.3	0.6	12.0	0.124	8.04	8.04
10	ED	4	0.3	0.8	7.47	0.0862	3.47	3.47
11	ED	4	0.6	0.6	10.1	0.151	6.08	6.09
12	ED	4	0.6	0.8	5.80	0.107	1.80	1.81
13	ED	5	0	0.6	16.0	0.102	11.0	11.0
14	ED	5	0	0.8	10.2	0.0490	5.20	5.20
15	ED	5	0.3	0.6	14.8	0.160	9.78	9.78
16	ED	5	0.3	0.8	9.10	0.111	4.10	4.10
17	ED	5	0.6	0.6	11.9	0.187	6.94	6.95
18	ED	5	0.6	0.8	6.74	0.126	1.74	1.75
19	ED	6	0	0.6	19.2	0.111	13.2	13.2
20	ED	6	0	0.8	12.2	0.0539	6.22	6.22
21	ED	6	0.3	0.6	17.4	0.194	11.4	11.4
22	ED	6	0.3	0.8	10.7	0.137	4.68	4.68
23	ED	6	0.6	0.6	13.7	0.238	7.69	7.69
24	ED	6	0.6	0.8	7.59	0.154	1.59	1.59
25	ED	7	0	0.6	22.3	0.122	15.3	15.3
26	ED	7	0	0.8	14.2	0.0580	7.24	7.24
27	ED	7	0.3	0.6	20.0	0.224	13.0	13.0
28	ED	7	0.3	0.8	12.2	0.162	5.23	5.23
29	ED	7	0.6	0.6	15.3	0.257	8.31	8.32
30	ED	7	0.6	0.8	8.38	0.180	1.38	1.39
31	EGA*	3	0	0.6	3	0	0	0
…								
61	MAP*	3	0	0.6	3	0	0	0
…								
91	PA*	3	0	0.6	3	0	0	0
…								

Note. Effective dimensionality (ED), exploratory graph análisis (EGA), Velicer’s mínimum average partial (MAP) and parallel analysis (PA) were evaluated across number of factors (n_factors), factor intercorrelations (rho), and reliability. Outcomes included mean estimates across resampling (mean_est), standard deviation (sd_est), mean estimates – n_factors (bias), and root mean squared error (rmse).
* Rows 31-120 are omitted; EGA, MAP, and PA recovered the correct number of factors across all simulated scenarios.

Supplementary Table S2. *Simulation 1: Effective dimensionality across hierarchical levels*

	N factors k	rho	Reliability	Mean ED			Mean SD		
				items	facets	domains	items	facets	domains
1	3	0	0.6	9.65	5.27	3.00	0.0811	0.0364	0.00228
2	3	0	0.8	6.14	4.11	3.00	0.0374	0.0181	0.00241
3	3	0.3	0.6	9.22	4.98	2.80	0.102	0.0554	0.0277
4	3	0.3	0.8	5.75	3.82	2.77	0.0628	0.0420	0.0299
5	3	0.6	0.6	8.06	4.22	2.29	0.119	0.0684	0.0381
6	3	0.6	0.8	4.76	3.09	2.21	0.0722	0.0506	0.0373
7	4	0	0.6	12.8	7.02	3.99	0.0909	0.0415	0.00342
8	4	0	0.8	8.17	5.47	3.99	0.0436	0.0208	0.00349
9	4	0.3	0.6	12.0	6.48	3.63	0.124	0.0724	0.0417
10	4	0.3	0.8	7.47	4.95	3.58	0.0862	0.0603	0.0450
11	4	0.6	0.6	10.1	5.19	2.78	0.151	0.0893	0.0517
12	4	0.6	0.8	5.80	3.74	2.65	0.107	0.0745	0.0559
13	5	0	0.6	16.0	8.77	4.99	0.102	0.0453	0.00465
14	5	0	0.8	10.2	6.83	4.99	0.0490	0.0229	0.00457
15	5	0.3	0.6	14.8	7.94	4.43	0.160	0.0943	0.0559
16	5	0.3	0.8	9.10	6.02	4.36	0.111	0.0787	0.0588
17	5	0.6	0.6	11.9	6.08	3.21	0.187	0.112	0.0671
18	5	0.6	0.8	6.74	4.31	3.03	0.126	0.0880	0.0660
19	6	0	0.6	19.2	10.5	5.99	0.111	0.0519	0.00549
20	6	0	0.8	12.2	8.19	5.99	0.0539	0.0251	0.00549
21	6	0.3	0.6	17.4	9.33	5.19	0.194	0.118	0.0703
22	6	0.3	0.8	10.7	7.05	5.09	0.137	0.0969	0.0730
23	6	0.6	0.6	13.7	6.89	3.60	0.238	0.140	0.0836
24	6	0.6	0.8	7.59	4.82	3.38	0.154	0.107	0.0805
25	7	0	0.6	22.3	12.3	6.98	0.122	0.0564	0.00634
26	7	0	0.8	14.2	9.55	6.98	0.0580	0.0284	0.00659
27	7	0.3	0.6	20.0	10.7	5.93	0.224	0.137	0.0829
28	7	0.3	0.8	12.2	8.06	5.81	0.162	0.114	0.0877
29	7	0.6	0.6	15.3	7.64	3.96	0.257	0.152	0.0904
30	7	0.6	0.8	8.38	5.30	3.69	0.180	0.125	0.0926

Note. Effective dimensionality (ED) is reported across hierarchical levels (items, facets, domains) under varying number of factors (k), inter-factor correlation (rho), and reliability. Values represent mean ED and standard deviation across simulation replications.

Supplementary Table S3. Simulation 1: Effective dimensionality under overfactoring conditions

	n_factors	rho	reliability	k	ED_mean	ED_sd
1	3	0	0.6	1	1	0
2	3	0	0.6	2	2.00	0.00223
3	3	0	0.6	3	3.00	0.00276
**4**	**3**	**0**	**0.6**	**4**	**3.88**	**0.225**
**5**	**3**	**0**	**0.6**	**5**	**4.70**	**0.360**
**6**	**3**	**0**	**0.6**	**6**	**5.41**	**0.459**
**7**	**3**	**0**	**0.6**	**7**	**6.01**	**0.542**
8	3	0	0.8	1	1	0
9	3	0	0.8	2	2.00	0.00215
10	3	0	0.8	3	3.00	0.00260
**11**	**3**	**0**	**0.8**	**4**	**3.90**	**0.263**
**12**	**3**	**0**	**0.8**	**5**	**4.66**	**0.460**
**13**	**3**	**0**	**0.8**	**6**	**5.36**	**0.616**
**14**	**3**	**0**	**0.8**	**7**	**5.95**	**0.726**
15	3	0.3	0.6	1	1	0
16	3	0.3	0.6	2	1.91	0.0252
17	3	0.3	0.6	3	2.76	0.0327
**18**	**3**	**0.3**	**0.6**	**4**	**3.67**	**0.211**
**19**	**3**	**0.3**	**0.6**	**5**	**4.46**	**0.364**
**20**	**3**	**0.3**	**0.6**	**6**	**5.17**	**0.456**
**21**	**3**	**0.3**	**0.6**	**7**	**5.79**	**0.541**
22	3	0.3	0.8	1	1	0
23	3	0.3	0.8	2	1.90	0.0248
24	3	0.3	0.8	3	2.76	0.0320
**25**	**3**	**0.3**	**0.8**	**4**	**3.65**	**0.273**
**26**	**3**	**0.3**	**0.8**	**5**	**4.44**	**0.454**
**27**	**3**	**0.3**	**0.8**	**6**	**5.13**	**0.620**
**28**	**3**	**0.3**	**0.8**	**7**	**5.69**	**0.737**
29	3	0.6	0.6	1	1	0
30	3	0.6	0.6	2	1.62	0.0387
31	3	0.6	0.6	3	2.17	0.0437
**32**	**3**	**0.6**	**0.6**	**4**	**3.08**	**0.195**
**33**	**3**	**0.6**	**0.6**	**5**	**3.88**	**0.338**
**34**	**3**	**0.6**	**0.6**	**6**	**4.56**	**0.441**
**35**	**3**	**0.6**	**0.6**	**7**	**5.18**	**0.530**
36	3	0.6	0.8	1	1	0
37	3	0.6	0.8	2	1.62	0.0366
38	3	0.6	0.8	3	2.16	0.0394
**39**	**3**	**0.6**	**0.8**	**4**	**3.04**	**0.242**
**40**	**3**	**0.6**	**0.8**	**5**	**3.82**	**0.423**
**41**	**3**	**0.6**	**0.8**	**6**	**4.51**	**0.589**
**42**	**3**	**0.6**	**0.8**	**7**	**5.07**	**0.698**

43	5	0	0.6	1	1	0
44	5	0	0.6	2	2.00	0.00537
45	5	0	0.6	3	2.99	0.00477
46	5	0	0.6	4	3.99	0.00420
47	5	0	0.6	5	4.99	0.00554
**48**	**5**	**0**	**0.6**	**6**	**5.97**	**0.0908**
**49**	**5**	**0**	**0.6**	**7**	**6.91**	**0.183**
**50**	**5**	**0**	**0.6**	**8**	**7.82**	**0.271**
**51**	**5**	**0**	**0.6**	**9**	**8.67**	**0.360**
52	5	0	0.8	1	1	0
53	5	0	0.8	2	2.00	0.00548
54	5	0	0.8	3	2.99	0.00455
55	5	0	0.8	4	3.99	0.00338
56	5	0	0.8	5	4.99	0.00489
**57**	**5**	**0**	**0.8**	**6**	**5.97**	**0.110**
**58**	**5**	**0**	**0.8**	**7**	**6.92**	**0.222**
**59**	**5**	**0**	**0.8**	**8**	**7.83**	**0.330**
**60**	**5**	**0**	**0.8**	**9**	**8.66**	**0.464**
61	5	0.3	0.6	1	1	0
62	5	0.3	0.6	2	1.88	0.0288
63	5	0.3	0.6	3	2.75	0.0444
64	5	0.3	0.6	4	3.58	0.0546
65	5	0.3	0.6	5	4.34	0.0631
**66**	**5**	**0.3**	**0.6**	**6**	**5.34**	**0.109**
**67**	**5**	**0.3**	**0.6**	**7**	**6.31**	**0.175**
**68**	**5**	**0.3**	**0.6**	**8**	**7.22**	**0.274**
**69**	**5**	**0.3**	**0.6**	**9**	**8.07**	**0.362**
70	5	0.3	0.8	1	1	0
71	5	0.3	0.8	2	1.88	0.0290
72	5	0.3	0.8	3	2.74	0.0451
73	5	0.3	0.8	4	3.57	0.0570
74	5	0.3	0.8	5	4.32	0.0615
**75**	**5**	**0.3**	**0.8**	**6**	**5.30**	**0.130**
**76**	**5**	**0.3**	**0.8**	**7**	**6.25**	**0.229**
**77**	**5**	**0.3**	**0.8**	**8**	**7.17**	**0.329**
**78**	**5**	**0.3**	**0.8**	**9**	**8.05**	**0.451**
79	5	0.6	0.6	1	1	0
80	5	0.6	0.6	2	1.58	0.0384
81	5	0.6	0.6	3	2.15	0.0817
82	5	0.6	0.6	4	2.66	0.109
83	5	0.6	0.6	5	3.03	0.0719
**84**	**5**	**0.6**	**0.6**	**6**	**4.01**	**0.0961**
**85**	**5**	**0.6**	**0.6**	**7**	**4.96**	**0.170**
**86**	**5**	**0.6**	**0.6**	**8**	**5.87**	**0.255**
**87**	**5**	**0.6**	**0.6**	**9**	**6.74**	**0.344**
88	5	0.6	0.8	1	1	0
89	5	0.6	0.8	2	1.57	0.0358
90	5	0.6	0.8	3	2.13	0.0727

91	5	0.6	0.8	4	2.61	0.106
92	5	0.6	0.8	5	2.95	0.0674
**93**	**5**	**0.6**	**0.8**	**6**	**3.88**	**0.101**
**94**	**5**	**0.6**	**0.8**	**7**	**4.80**	**0.194**
**95**	**5**	**0.6**	**0.8**	**8**	**5.70**	**0.299**
**96**	**5**	**0.6**	**0.8**	**9**	**6.54**	**0.437**
97	7	0	0.6	1	1	0
98	7	0	0.6	2	1.99	0.00741
99	7	0	0.6	3	2.99	0.00673
100	7	0	0.6	4	3.99	0.00586
101	7	0	0.6	5	4.99	0.00528
102	7	0	0.6	6	5.98	0.00553
103	7	0	0.6	7	6.97	0.00744
**104**	**7**	**0**	**0.6**	**8**	**7.97**	**0.0399**
**105**	**7**	**0**	**0.6**	**9**	**8.96**	**0.0616**
**106**	**7**	**0**	**0.6**	**10**	**9.92**	**0.125**
**107**	**7**	**0**	**0.6**	**11**	**10.9**	**0.189**
108	7	0	0.8	1	1	0
109	7	0	0.8	2	1.99	0.00674
110	7	0	0.8	3	2.99	0.00644
111	7	0	0.8	4	3.99	0.00578
112	7	0	0.8	5	4.99	0.00444
113	7	0	0.8	6	5.99	0.00468
114	7	0	0.8	7	6.98	0.00702
**115**	**7**	**0**	**0.8**	**8**	**7.97**	**0.0373**
**116**	**7**	**0**	**0.8**	**9**	**8.96**	**0.0865**
**117**	**7**	**0**	**0.8**	**10**	**9.93**	**0.160**
**118**	**7**	**0**	**0.8**	**11**	**10.9**	**0.233**
119	7	0.3	0.6	1	1	0
120	7	0.3	0.6	2	1.85	0.0339
121	7	0.3	0.6	3	2.72	0.0471
122	7	0.3	0.6	4	3.57	0.0615
123	7	0.3	0.6	5	4.39	0.0791
124	7	0.3	0.6	6	5.16	0.0984
125	7	0.3	0.6	7	5.82	0.0884
**126**	**7**	**0.3**	**0.6**	**8**	**6.84**	**0.0884**
**127**	**7**	**0.3**	**0.6**	**9**	**7.86**	**0.106**
**128**	**7**	**0.3**	**0.6**	**10**	**8.86**	**0.143**
**129**	**7**	**0.3**	**0.6**	**11**	**9.83**	**0.198**
130	7	0.3	0.8	1	1	0
131	7	0.3	0.8	2	1.85	0.0352
132	7	0.3	0.8	3	2.71	0.0467
133	7	0.3	0.8	4	3.56	0.0613
134	7	0.3	0.8	5	4.37	0.0772
135	7	0.3	0.8	6	5.12	0.0955
136	7	0.3	0.8	7	5.77	0.0899
**137**	**7**	**0.3**	**0.8**	**8**	**6.77**	**0.0928**
**138**	**7**	**0.3**	**0.8**	**9**	**7.76**	**0.112**

**139**	**7**	**0.3**	**0.8**	**10**	**8.74**	**0.162**
**140**	**7**	**0.3**	**0.8**	**11**	**9.71**	**0.227**
141	7	0.6	0.6	1	1	0
142	7	0.6	0.6	2	1.54	0.0487
143	7	0.6	0.6	3	2.14	0.0969
144	7	0.6	0.6	4	2.71	0.135
145	7	0.6	0.6	5	3.23	0.172
146	7	0.6	0.6	6	3.65	0.205
147	7	0.6	0.6	7	3.84	0.0889
**148**	**7**	**0.6**	**0.6**	**8**	**4.82**	**0.0952**
**149**	**7**	**0.6**	**0.6**	**9**	**5.82**	**0.114**
**150**	**7**	**0.6**	**0.6**	**10**	**6.82**	**0.144**
**151**	**7**	**0.6**	**0.6**	**11**	**7.81**	**0.189**
152	7	0.6	0.8	1	1	0
153	7	0.6	0.8	2	1.54	0.0450
154	7	0.6	0.8	3	2.11	0.0980
155	7	0.6	0.8	4	2.65	0.142
156	7	0.6	0.8	5	3.13	0.179
157	7	0.6	0.8	6	3.49	0.206
158	7	0.6	0.8	7	3.63	0.0930
**159**	**7**	**0.6**	**0.8**	**8**	**4.54**	**0.101**
**160**	**7**	**0.6**	**0.8**	**9**	**5.47**	**0.117**
**161**	**7**	**0.6**	**0.8**	**10**	**6.41**	**0.149**
**162**	**7**	**0.6**	**0.8**	**11**	**7.35**	**0.205**

Note. Effective dimensionality (ED) is reported as a function of the number of extracted factors (k) under varying number of true factors (n_factors), inter-factor correlation (rho), and reliability. Values represent mean ED and standard deviation across simulation replications. Values where k > n_factors correspond to overfactoring conditions and are indicated in bold.

Supplementary Table S4. *Sensitivity Analysis of effective dimensionality (ED) estimates: disattenuated ED and polychoric/tetrachoric-based ED.*

Scales	ED	Disattenuated ED	Poly/tetrachoric-based ED
PID-5-SF (n=7,953)			
5 domains	3.46 [3.41, 3.52]	3.22 [3.16, 3.27]	
25 facets	12.47 [12.22, 12.69]	8.82 [8.55, 9.04]	
100 items	43.58 [42.53, 44.18]	—	30.65
PiCD (n=7,566)			
5 domains	4.08 [4.04, 4.11]	3.79 [3.74, 3.83]	
60 items	35.35 [34.82, 35.63]	—	29.46
DAPP-BQ (n=4,185)			
4 domains	3.38 [3.34, 3.41]	3.33 [3.28, 3.37]	
18 facets	8.22 [8.03, 8.40]	6.87 [6.69, 7.05]	
72 subfacets	24.49 [23.70, 24.99]	12.02 [11.42, 12.41]	
290 items	103.63 [98.00, 103.1]	—	72.02
PDQ-4+ (n=2,510)			
3 clusters	2.25 [2.20, 2.30]	1.93 [1.86, 2.00]	
12 disorders	7.43 [7.22, 7.62]	3.49 [3.25, 3.73]	
92 items	66.36 [64.22, 66.17]	—	40.41
TCI-R-140 (n=5,176)			
7 dimensions	5.97 [5.92, 6.01]	5.62 [5.55, 5.67]	
29 facets	18.04 [17.80, 18.19]	9.51 [9.21, 9.73]	
140 items	82.78 [80.89, 83.74]	—	69.33

Note. Effective dimensionality (ED) is compared across alternative specifications: disattenuated ED for facets and domains (ω_t is not applicable at the item-level) and ED based on polychoric/tetrachoric correlations for Likert/binary items. ED estimates based on polychoric (PID-5, PiCD, DAPP-BQ and TCI-R) and tetrachoric (PDQ-4+) correlations lack confidence intervals due to computational constraints, and should be interpreted with caution. PID-5-SF = Personality Inventory for DSM-5-Short Form; PiCD = Personality Inventory for ICD-11; DAPP-BQ = Dimensional Assessment of PersonalityPathology – Basic Questionnaire; PDQ-4+ = Personality Diagnostic Questionnaire-4+; TCI-R = Temperament and Character Inventory-Revised.

Supplementary Table S5. *Pairwise sample sizes and individual vs. joint effective dimensionality (ED) estimates across personality questionnaires.*

	PID-5-SF<br>ED [95% CI]	PiCD<br>ED [95% CI]	DAPP-BQ<br>ED [95% CI]	PDQ-4+<br>ED [95% CI]	TCI-R<br>ED [95% CI]
PID-5-SF	—<br>(n = 7,953)				
PiCD	PID-5: **3.48** [3.42, 3.55]<br>PiCD: **4.07** [4.02, 4.12]<br>**5.69** [5.59, 5.80]<br>(n=4,243)	—<br>(n = 7,566)			
DAPP-BQ	PID-5: **3.90** [3.76, 4.01]<br>DAPP: **3.52** [3.45, 3.58]<br>**5.33** [5.14, 5.50]<br>(n=1,000)	PiCD: **4.30** [4.15, 4.40]<br>DAPP: **3.55** [3.43, 3.64]<br>**5.81** [5.50, 6.02]<br>(n=405)	—<br>(n = 4,185)		
PDQ-4+	—<br>(n=0)	—<br>(n=0)	DAPP: **3.49** [3.41, 3.55]<br>PDQ: **2.27** [2.19, 2.34]<br>**4.32** [4.18, 4.45]<br>(n=1,018)	—<br>(n = 2,510)	
TCI-R	PID-5: **3.53** [3.44, 3.61]<br>TCI-R: **5.99** [5.93, 6.04]<br>**7.78** [7.64, 7.90]<br>(n=3,162)	PiCD: **4.14** [4.08, 4.19]<br>TCI-R: **5.97** [5.90, 6.02]<br>**8.08** [7.95, 8.18]<br>(n=2,692)	DAPP: **3.50** [3.44, 3.56]<br>TCI-R: **5.88** [5.78, 5.96]<br>**6.94** [6.79, 7.06]<br>(n=1,236)	PDQ: **2.29** [2.21, 2.38]<br>TCI-R: **5.85** [5.73, 5.94]<br>**6.71** [6.51, 6.89]<br>(n=858)	—<br>(n = 5,176)

Note. Each cell displays instrument-specific ED values, joint ED, and sample size (n). Not all possible combinations were available in the dataset. PID-5-SF = Personality Inventory for DSM-5-Short Form; PiCD = Personality Inventory for ICD-11; DAPP-BQ = Dimensional Assessment of Personality Pathology – Basic Questionnaire; PDQ-4+ = Personality Diagnostic Questionnaire-4+; TCI-R = Temperament and Character Inventory-Revised.

Supplementary Table S6. *Effective dimensionality (ED) and redundancy across hierarchical levels in community and clinical samples*.

	Community sample		Clinical sample		
Scales	ED [95% CI]	Redun-dancy (%)	ED [95% CI]	Redun-dancy (%)	Difference [95% CI]
PID-5-SF (n=7,953)					
5 domains	3.66 [3.60, 3.72]	26.8%	3.62 [3.54, 3.70]	27.6%	-.04 [-.14, .07]
25 facets	13.49 [13.18, 13.76]	46.0%	13.10 [12.69, 13.43]	47.6%	-.39 [-.87, .08]
100 items	50.02 [48.60, 50. 61]	50.0%	42.80 [40.97, 43.41]	57.2%	**-7.22 [-8.96, -5.85]**
PiCD (n=7,566)					
5 domains	4.19 [4.15, 4.22]	16.2%	4.11 [4.03, 4.17]	17.8%	-.08 [-.16, .00]
60 items	37.16 [36.54, 37.43]	38.1%	35.78 [34.37, 36.00]	40.4%	**-1.38 [-2.66, -.89]**
DAPP-BQ (n=4,185)					
4 domains	3.41 [3.36, 3.46]	14.8%	3.51 [3.46, 3.55]	12.3%	**.10 [.02, .16]**
18 facets	8.67 [8.39, 8.91]	51.8%	9.14 [8.86, 9.38]	49.2%	**.47 [.10, .84]**
72 subfacets	28.01 [26.68, 28.66]	61.1%	26.40 [25.17, 26.91]	63.3%	**-1.61 [-2.94, -.22]**
290 items	125.78 [114.74, 121.06]	56.6%	101.88 [92.85, 99.14]	64.9%	**-23.90 [-27.21, -.17.36]**
PDQ-4+ (n=2,510)					
3 clusters	2.34 [2.25, 2.43]	22.0%	2.26 [2.20, 2.32]	24.7%	-.08 [-.19, .02]
12 disorders	8.35 [7.97, 8.64]	30.4%	7.29 [7.05, 7.52]	39.3%	**-1.06 [-1.43, -.63]**
92 items	70.09 [64.70, 67.62]	23.8%	64.63 [61.78, 64.17]	29.8%	**-5.46 [-5.09, -1.29]**
TCI-R-140 (n=5,176)					
7 dimensions	5.98 [5.92, 6.03]	14.6%	5.97 [5.89, 6.02]	14.7%	-.01 [-.11, .07]
29 facets	18.95 [18.62, 19.14]	34.7%	17.82 [17.36, 18.05]	38.6%	**-1.13 [-1.60, -.76]**
140 items	87.21 [84.37, 86.55]	37.7%	78.95 [75.31, 77.88]	43.6%	**-8.26 [-10.52, -7.17]**

Note. Significant differences are in bold type; positive values indicate higher effective dimensionality (i.e., lower redundancy) in the clinical sample. PID-5-SF = Personality Inventory for DSM-5-Short Form; PiCD = Personality Inventory for ICD-11; DAPP-BQ = Dimensional Assessment of PersonalityPathology – Basic Questionnaire; PDQ-4+ = Personality Diagnostic Questionnaire-4+; TCI-R = Temperament and Character Inventory-Revised.

Supplementary Table S7. *Sensitivity analyses of predictive performance across hierarchical levels: OLS (raw and residualized traits) and ridge and Poisson/ordinal regressions for non-continuous outcomes.*

	OLS regression (R^2)					Regression non-continuous outcomes (R^2)	
	Raw traits			Residualized traits			
Scales	In-sample	cross-validated	ΔR² vs. lower level [95% CI]	In-sample	cross-validated	Ridge	Poisson and ordinal
PID-5-SF (n=7,953)							
5 domains	.06	.06	—	—	—	.05 [.01, .11]	.04 [.02, .07]
25 facets	.10	.09	.03 [-.02, .08]	.05	.04	.09 [.03, .16]	.07 [.04, .12]
100 items	.17	.10	.01 [-.04, .06]	.07	.02	.11 [.05, .19]	.08 [.05, .13]
PiCD (n=7,566)							
5 domains	.05	.05	—	—	—	.05 [.01, .12]	.05 [.02, .09]
60 items	.16	.09	.04 [-.03, .12]	.11	.05	.09 [.02, .19]	.07 [.04, .13]
DAPP-BQ (n=4,185)							
4 domains	.06	.07	—	—	—	.06 [.00, .19]	.04 [.00, .10]
18 facets	.13	.09	.02 [-.08, .13]	.07	.05	.09 [.00, .23]	.07 [.01, .16]
72 subfacets	.27	.08	.00 [-.12, .11]	.18	.03	.10 [.01, .26]	.06 [.01, .15]
290 items	.65	.05	-.03 [-.18, .10]	.38	.03	.12 [.02, .26]	.06 [.01, .14]
PDQ-4+ (n=2,510)							
3 clusters	.06	.07	—	—	—	.08 [.01, .18]	.05 [.01, .11]
12 disorders	.09	.08	.01 [-.06, .07]	.03	.03	.09 [.01, .19]	.06 [.01, .13]
92 items	.25	.09	.01 [-.09, .11]	.16	.03	.12 [.02, .24]	.08 [.03, .16]
TCI-R-140 (n=5,176)							
7 dimensions	.06	.06	—	—	—	.06 [.01, .14]	.05 [.02, .08]
29 facets	.13	.09	.03 [-.05, .12]	.07	.05	.09 [.03, .18]	.07 [.03, .12]
140 items	.27	.10	.00 [-.10, .08]	.14	.03	.12 [.05, .22]	.08 [.04, .14]

Note. ED = effective dimensionality. ΔR² = incremental variance explained relative to the immediately lower level. Residualized traits = scores after partialling out the higher level. Regression non-continuous outcomes (R^2) compares ridge and Poisson/ordinal regression applied to three count (lifetime number of romantic partners, number of children, and number of close friends) and two ordinal outcomes (academic degree and job category). PID-5-SF = Personality Inventory for DSM-5-Short Form; PiCD = Personality Inventory for ICD-11; DAPP-BQ = Dimensional Assessment of Personality Pathology – Basic Questionnaire; PDQ-4+ = Personality Diagnostic Questionnaire-4+; TCI-R = Temperament and Character Inventory-Revised.

Supplementary Table S8. *Ratio of effective dimensionality vs. nominal dimensionality (ED/k) across 62 solutions per instrument, varying by rotation method.*

Nominal number of factors	Simpli-max	Biquar-timin	Obl CF-Equamax	Geo-minQ	Oblimin	Promax	BentlerQ
**PID-5-SF**							
2	0,87	—	**0,93**	0,90	0,89	0,81	0,87
3	**1,00**	**1,00**	**0,90**	**0,92**	0,88	0,77	0,77
4	**1,00**	**0,99**	0,87	0,84	0,82	0,71	0,73
**5 (theory)**	**0,98**	**0,99**	0,85	0,86	0,81	0,72	0,61
6	**0,98**	**0,97**	0,84	0,84	0,81	0,74	0,69
7	**0,99**	**0,96**	0,83	0,84	0,79	0,75	0,61
8	**0,99**	**0,94**	0,80	0,75	0,76	0,72	0,56
9	**1,00**	**0,92**	0,79	0,76	0,75	0,71	0,55
10	**1,00**	0,90	0,78	0,71	0,74	0,68	0,51
**PiCD**							
2	**1,00**	—	**0,99**	**0,97**	**0,98**	**0,98**	**0,97**
3	**0,93**	**0,98**	**0,98**	**0,95**	**0,96**	**0,92**	**0,94**
4	**0,95**	**0,99**	**0,97**	**0,94**	**0,96**	0,89	**0,90**
**5 (theory)**	**0,97**	**0,94**	**0,94**	0,89	**0,91**	0,83	0,83
6	**0,96**	**0,94**	**0,92**	0,88	0,89	0,81	0,80
7	**0,99**	**0,93**	**0,91**	0,87	0,88	0,78	0,80
8	**0,98**	**0,90**	0,89	0,84	0,84	0,75	0,74
9	**0,99**	**0,90**	0,87	0,83	0,84	0,74	0,71
10	**0,99**	0,88	0,86	0,82	0,82	0,72	0,65
**DAPP-BQ**							
2	**1,00**	—	**0,92**	0,88	0,87	0,83	0,83
3	**0,95**	**1,00**	**0,90**	**0,90**	**0,90**	0,79	0,84
**4 (theory)**	**0,94**	**0,99**	0,89	**0,92**	**0,92**	0,86	0,85
5	**0,97**	**0,99**	0,87	**0,92**	0,87	0,83	0,76
6	**0,98**	**0,94**	0,84	0,89	0,82	0,80	0,69
7	**0,99**	**0,93**	0,80	0,87	0,76	0,71	0,57
8	**1,00**	**0,91**	0,79	0,81	0,75	0,71	0,57
9	**1,00**	0,87	0,76	0,82	0,72	0,72	0,52
10	**1,00**	0,87	0,74	0,67	0,70	0,71	0,57
**PDQ-4+**							
2	**1,00**	—	0,90	0,85	0,84	0,76	0,80
**3 (theory)**	**1,00**	**1,00**	0,87	0,84	0,83	0,72	0,76
4	**1,00**	**0,98**	0,84	0,79	0,79	0,67	0,70
5	**0,99**	**0,95**	0,80	0,72	0,74	0,60	0,62
6	**0,99**	**0,94**	0,77	0,70	0,71	0,55	0,57

7	**0,99**	**0,94**	0,77	0,72	0,72	0,61	0,64
8	**1,00**	0,89	0,68	0,61	0,63	0,53	0,50
9	**1,00**	0,86	0,66	0,60	0,64	0,53	0,30
10	**1,00**	0,81	0,60	0,59	0,57	0,52	0,44
**TCI-R**							
2	**1,00**	—	**1,00**	**1,00**	**1,00**	**1,00**	**0,99**
3	**0,99**	**1,00**	**0,99**	**0,98**	**0,99**	**0,97**	**0,99**
4	**0,97**	**0,99**	**0,98**	**0,95**	**0,97**	**0,95**	**0,92**
5	**0,97**	**0,96**	**0,96**	**0,95**	**0,95**	0,90	0,89
6	**0,98**	**0,94**	**0,94**	**0,92**	**0,92**	0,86	0,81
**7 (theory)**	**0,99**	**0,93**	**0,93**	**0,90**	**0,91**	0,83	0,77
8	**1,00**	**0,91**	**0,90**	0,86	0,87	0,80	0,78
9	**1,00**	0,89	0,89	0,84	0,85	0,78	0,75
10	**1,00**	0,87	0,87	0,82	0,83	0,76	0,72
$\text{Mean}_{\text{(unweighted)}}$	0,99	0,94	0,87	0,84	0,84	0,77	0,72
SD	0,04	0,07	0,09	0,11	0,10	0,12	0,15

Note. Biquartimin rotation requires a minimum of three factors to be identified. Solutions with ED/*k* ratio within 0.90–1.10 are shaded in blue. The number of factors suggested by theory is shown on the left column. PID-5-SF = Personality Inventory for DSM-5-Short Form; PiCD = Personality Inventory for ICD-11; DAPP-BQ = Dimensional Assessment of Personality Pathology – Basic Questionnaire; PDQ-4+ = Personality Diagnostic Questionnaire-4+; TCI-R = Temperament and Character Inventory-Revised.

Supplementary Table S9. *Simulation 2: Classification performance and calibration metrics at T = 70 by decision rule across thresholds.*

	rule	N traits	rho	Distri-bution	Skew param	Sensi-tivity	Speci-ficity	Preci-sion	Jaccard	caseness rate	target	Infla-tion
1	any	5	0	normal	0	0.619	0.903	0.129	0.119	0.109	0.0228	4.77
2	any	5	0	skewed	0.5	0.587	0.901	0.25	0.213	0.125	0.0228	5.46
3	any	5	0	skewed	1	0.558	0.924	0.345	0.271	0.109	0.0228	4.77
4	any	5	0.2	normal	0	0.546	0.909	0.136	0.122	0.103	0.0228	4.52
5	any	5	0.2	skewed	0.5	0.532	0.908	0.258	0.21	0.117	0.0228	5.12
6	any	5	0.2	skewed	1	0.516	0.929	0.356	0.267	0.102	0.0228	4.49
7	any	5	0.4	normal	0	0.454	0.918	0.166	0.137	0.095	0.0228	4.17
8	any	5	0.4	skewed	0.5	0.494	0.922	0.312	0.236	0.106	0.0228	4.65
9	any	5	0.4	skewed	1	0.499	0.943	0.429	0.3	0.092	0.0228	4.06
10	any	5	0.6	normal	0	0.355	0.932	0.24	0.157	0.085	0.0228	3.73
11	any	5	0.6	skewed	0.5	0.442	0.942	0.429	0.275	0.092	0.0228	4.04
12	any	5	0.6	skewed	1	0.468	0.962	0.574	0.346	0.08	0.0228	3.5
13	any	10	0	normal	0	0.729	0.807	0.08	0.078	0.206	0.0228	9.02
14	any	10	0	skewed	0.5	0.68	0.796	0.179	0.165	0.234	0.0228	10.2
15	any	10	0	skewed	1	0.626	0.834	0.264	0.228	0.205	0.0228	9.01
16	any	10	0.2	normal	0	0.615	0.827	0.095	0.089	0.185	0.0228	8.13
17	any	10	0.2	skewed	0.5	0.571	0.821	0.192	0.168	0.207	0.0228	9.06
18	any	10	0.2	skewed	1	0.54	0.856	0.282	0.227	0.182	0.0228	7.97
19	any	10	0.4	normal	0	0.493	0.854	0.136	0.118	0.162	0.0228	7.1
20	any	10	0.4	skewed	0.5	0.511	0.857	0.26	0.208	0.176	0.0228	7.71
21	any	10	0.4	skewed	1	0.507	0.89	0.366	0.27	0.154	0.0228	6.76
22	any	10	0.6	normal	0	0.364	0.887	0.237	0.153	0.136	0.0228	5.96
23	any	10	0.6	skewed	0.5	0.454	0.904	0.419	0.272	0.143	0.0228	6.28
24	any	10	0.6	skewed	1	0.475	0.935	0.546	0.338	0.124	0.0228	5.43
25	any	20	0	normal	0	0.847	0.642	0.051	0.051	0.369	0.0228	16.2
26	any	20	0	skewed	0.5	0.8	0.616	0.133	0.129	0.413	0.0228	18.1
27	any	20	0	skewed	1	0.725	0.674	0.212	0.196	0.369	0.0228	16.2
28	any	20	0.2	normal	0	0.688	0.703	0.072	0.07	0.31	0.0228	13.6
29	any	20	0.2	skewed	0.5	0.616	0.688	0.155	0.141	0.338	0.0228	14.8

30	any	20	0.2	skewed	1	0.567	0.736	0.234	0.198	0.301	0.0228	13.2
31	any	20	0.4	normal	0	0.535	0.764	0.129	0.114	0.255	0.0228	11.2
32	any	20	0.4	skewed	0.5	0.528	0.766	0.237	0.195	0.27	0.0228	11.8
33	any	20	0.4	skewed	1	0.513	0.81	0.322	0.247	0.238	0.0228	10.4
34	any	20	0.6	normal	0	0.385	0.83	0.272	0.168	0.203	0.0228	8.91
35	any	20	0.6	skewed	0.5	0.465	0.856	0.438	0.281	0.207	0.0228	9.1
36	any	20	0.6	skewed	1	0.48	0.895	0.528	0.332	0.18	0.0228	7.89
37	any	30	0	normal	0	0.906	0.511	0.041	0.041	0.498	0.0228	21.8
38	any	30	0	skewed	0.5	0.872	0.475	0.115	0.113	0.55	0.0228	24.1
39	any	30	0	skewed	1	0.797	0.542	0.193	0.184	0.499	0.0228	21.9
40	any	30	0.2	normal	0	0.733	0.611	0.066	0.064	0.402	0.0228	17.6
41	any	30	0.2	skewed	0.5	0.64	0.591	0.142	0.132	0.431	0.0228	18.9
42	any	30	0.2	skewed	1	0.586	0.645	0.217	0.188	0.388	0.0228	17.0
43	any	30	0.4	normal	0	0.565	0.7	0.136	0.12	0.321	0.0228	14.1
44	any	30	0.4	skewed	0.5	0.536	0.701	0.233	0.193	0.334	0.0228	14.6
45	any	30	0.4	skewed	1	0.518	0.75	0.305	0.238	0.297	0.0228	13.0
46	any	30	0.6	normal	0	0.4	0.792	0.312	0.186	0.249	0.0228	10.9
47	any	30	0.6	skewed	0.5	0.472	0.824	0.461	0.292	0.25	0.0228	11.0
48	any	30	0.6	skewed	1	0.483	0.865	0.521	0.329	0.217	0.0228	9.54
49	any	50	0	normal	0	0.962	0.323	0.032	0.032	0.683	0.0228	30.0
50	any	50	0	skewed	0.5	0.944	0.282	0.098	0.098	0.735	0.0228	32.2
51	any	50	0	skewed	1	0.886	0.348	0.177	0.173	0.683	0.0228	30.0
52	any	50	0.2	normal	0	0.788	0.483	0.062	0.061	0.528	0.0228	23.2
53	any	50	0.2	skewed	0.5	0.669	0.459	0.134	0.126	0.556	0.0228	24.4
54	any	50	0.2	skewed	1	0.612	0.513	0.205	0.181	0.508	0.0228	22.3
55	any	50	0.4	normal	0	0.605	0.613	0.156	0.137	0.41	0.0228	18
56	any	50	0.4	skewed	0.5	0.548	0.608	0.239	0.199	0.422	0.0228	18.5
57	any	50	0.4	skewed	1	0.523	0.66	0.289	0.228	0.378	0.0228	16.6
58	any	50	0.6	normal	0	0.424	0.74	0.381	0.219	0.311	0.0228	13.6
59	any	50	0.6	skewed	0.5	0.482	0.777	0.498	0.308	0.309	0.0228	13.6
60	any	50	0.6	skewed	1	0.489	0.82	0.515	0.327	0.27	0.0228	11.8
61	any	70	0	normal	0	0.984	0.204	0.027	0.027	0.8	0.0228	35.1
62	any	70	0	skewed	0.5	0.974	0.167	0.091	0.09	0.844	0.0228	37.0
63	any	70	0	skewed	1	0.935	0.223	0.172	0.17	0.8	0.0228	35.1
64	any	70	0.2	normal	0	0.823	0.398	0.063	0.062	0.612	0.0228	26.9

65	any	70	0.2	skewed	0.5	0.688	0.371	0.135	0.127	0.637	0.0228	27.9
66	any	70	0.2	skewed	1	0.632	0.422	0.202	0.181	0.589	0.0228	25.8
67	any	70	0.4	normal	0	0.636	0.554	0.18	0.158	0.472	0.0228	20.7
68	any	70	0.4	skewed	0.5	0.556	0.541	0.248	0.207	0.481	0.0228	21.1
69	any	70	0.4	skewed	1	0.528	0.594	0.281	0.225	0.434	0.0228	19.0
70	any	70	0.6	normal	0	0.445	0.705	0.439	0.249	0.354	0.0228	15.5
71	any	70	0.6	skewed	0.5	0.49	0.743	0.524	0.322	0.35	0.0228	15.4
72	any	70	0.6	skewed	1	0.492	0.785	0.514	0.327	0.307	0.0228	13.5
73	nominal	5	0	normal	0	0.362	0.985	0.363	0.222	0.023	0.0228	0.99
74	nominal	5	0	skewed	0.5	0.482	0.974	0.514	0.331	0.05	0.0228	2.19
75	nominal	5	0	skewed	1	0.511	0.975	0.597	0.38	0.058	0.0228	2.53
76	nominal	5	0.2	normal	0	0.321	0.986	0.37	0.207	0.022	0.0228	0.97
77	nominal	5	0.2	skewed	0.5	0.452	0.977	0.538	0.326	0.048	0.0228	2.1
78	nominal	5	0.2	skewed	1	0.486	0.978	0.63	0.378	0.055	0.0228	2.4
79	nominal	5	0.4	normal	0	0.251	0.987	0.401	0.178	0.021	0.0228	0.93
80	nominal	5	0.4	skewed	0.5	0.414	0.982	0.625	0.33	0.044	0.0228	1.94
81	nominal	5	0.4	skewed	1	0.465	0.985	0.737	0.398	0.05	0.0228	2.21
82	nominal	5	0.6	normal	0	0.178	0.989	0.475	0.138	0.019	0.0228	0.85
83	nominal	5	0.6	skewed	0.5	0.338	0.99	0.757	0.3	0.039	0.0228	1.69
84	nominal	5	0.6	skewed	1	0.397	0.994	0.881	0.375	0.044	0.0228	1.91
85	nominal	10	0	normal	0	0.289	0.984	0.291	0.17	0.022	0.0228	0.98
86	nominal	10	0	skewed	0.5	0.489	0.958	0.433	0.299	0.07	0.0228	3.06
87	nominal	10	0	skewed	1	0.535	0.951	0.513	0.355	0.091	0.0228	3.99
88	nominal	10	0.2	normal	0	0.241	0.984	0.309	0.156	0.022	0.0228	0.97
89	nominal	10	0.2	skewed	0.5	0.443	0.963	0.478	0.298	0.065	0.0228	2.85
90	nominal	10	0.2	skewed	1	0.493	0.96	0.568	0.358	0.083	0.0228	3.64
91	nominal	10	0.4	normal	0	0.177	0.987	0.365	0.129	0.02	0.0228	0.89
92	nominal	10	0.4	skewed	0.5	0.394	0.976	0.619	0.313	0.057	0.0228	2.49
93	nominal	10	0.4	skewed	1	0.463	0.977	0.72	0.391	0.072	0.0228	3.16
94	nominal	10	0.6	normal	0	0.115	0.99	0.473	0.091	0.018	0.0228	0.79
95	nominal	10	0.6	skewed	0.5	0.307	0.99	0.813	0.28	0.046	0.0228	2.03
96	nominal	10	0.6	skewed	1	0.378	0.994	0.911	0.361	0.057	0.0228	2.51
97	nominal	20	0	normal	0	0.22	0.982	0.22	0.124	0.022	0.0228	0.98
98	nominal	20	0	skewed	0.5	0.51	0.931	0.355	0.265	0.1	0.0228	4.38
99	nominal	20	0	skewed	1	0.576	0.907	0.432	0.327	0.145	0.0228	6.37

100	nominal	20	0.2	normal	0	0.174	0.984	0.257	0.114	0.021	0.0228	0.94
101	nominal	20	0.2	skewed	0.5	0.439	0.944	0.427	0.275	0.089	0.0228	3.89
102	nominal	20	0.2	skewed	1	0.502	0.928	0.502	0.335	0.125	0.0228	5.5
103	nominal	20	0.4	normal	0	0.12	0.986	0.338	0.089	0.02	0.0228	0.86
104	nominal	20	0.4	skewed	0.5	0.381	0.968	0.624	0.303	0.073	0.0228	3.2
105	nominal	20	0.4	skewed	1	0.461	0.963	0.695	0.38	0.1	0.0228	4.4
106	nominal	20	0.6	normal	0	0.073	0.991	0.483	0.059	0.017	0.0228	0.73
107	nominal	20	0.6	skewed	0.5	0.278	0.991	0.867	0.259	0.055	0.0228	2.4
108	nominal	20	0.6	skewed	1	0.362	0.992	0.918	0.345	0.074	0.0228	3.24
109	nominal	30	0	normal	0	0.189	0.981	0.189	0.104	0.022	0.0228	0.98
110	nominal	30	0	skewed	0.5	0.531	0.908	0.313	0.245	0.124	0.0228	5.44
111	nominal	30	0	skewed	1	0.611	0.867	0.389	0.312	0.191	0.0228	8.36
112	nominal	30	0.2	normal	0	0.141	0.983	0.231	0.094	0.021	0.0228	0.94
113	nominal	30	0.2	skewed	0.5	0.44	0.929	0.402	0.264	0.107	0.0228	4.68
114	nominal	30	0.2	skewed	1	0.51	0.901	0.47	0.323	0.158	0.0228	6.92
115	nominal	30	0.4	normal	0	0.096	0.986	0.336	0.073	0.019	0.0228	0.84
116	nominal	30	0.4	skewed	0.5	0.375	0.963	0.635	0.298	0.084	0.0228	3.68
117	nominal	30	0.4	skewed	1	0.464	0.951	0.678	0.375	0.121	0.0228	5.31
118	nominal	30	0.6	normal	0	0.055	0.991	0.503	0.045	0.016	0.0228	0.69
119	nominal	30	0.6	skewed	0.5	0.264	0.992	0.895	0.248	0.06	0.0228	2.64
120	nominal	30	0.6	skewed	1	0.354	0.99	0.914	0.336	0.085	0.0228	3.72
121	nominal	50	0	normal	0	0.151	0.981	0.153	0.082	0.022	0.0228	0.97
122	nominal	50	0	skewed	0.5	0.564	0.87	0.266	0.221	0.164	0.0228	7.18
123	nominal	50	0	skewed	1	0.666	0.796	0.343	0.293	0.267	0.0228	11.7
124	nominal	50	0.2	normal	0	0.111	0.983	0.21	0.075	0.021	0.0228	0.93
125	nominal	50	0.2	skewed	0.5	0.445	0.905	0.379	0.254	0.134	0.0228	5.87
126	nominal	50	0.2	skewed	1	0.519	0.856	0.431	0.307	0.208	0.0228	9.11
127	nominal	50	0.4	normal	0	0.071	0.987	0.341	0.055	0.019	0.0228	0.81
128	nominal	50	0.4	skewed	0.5	0.369	0.956	0.653	0.294	0.1	0.0228	4.38
129	nominal	50	0.4	skewed	1	0.466	0.932	0.652	0.366	0.151	0.0228	6.61
130	nominal	50	0.6	normal	0	0.04	0.992	0.537	0.033	0.015	0.0228	0.64
131	nominal	50	0.6	skewed	0.5	0.247	0.992	0.916	0.234	0.068	0.0228	2.97
132	nominal	50	0.6	skewed	1	0.349	0.986	0.903	0.328	0.1	0.0228	4.4
133	nominal	70	0	normal	0	0.13	0.98	0.13	0.07	0.022	0.0228	0.97
134	nominal	70	0	skewed	0.5	0.59	0.837	0.238	0.205	0.197	0.0228	8.63

135	nominal	70	0	skewed	1	0.708	0.734	0.317	0.28	0.331	0.0228	14.5
136	nominal	70	0.2	normal	0	0.094	0.982	0.201	0.065	0.021	0.0228	0.92
137	nominal	70	0.2	skewed	0.5	0.45	0.887	0.372	0.251	0.154	0.0228	6.78
138	nominal	70	0.2	skewed	1	0.527	0.819	0.409	0.298	0.246	0.0228	10.8
139	nominal	70	0.4	normal	0	0.06	0.987	0.354	0.047	0.018	0.0228	0.79
140	nominal	70	0.4	skewed	0.5	0.367	0.951	0.668	0.293	0.112	0.0228	4.89
141	nominal	70	0.4	skewed	1	0.469	0.916	0.637	0.362	0.173	0.0228	7.57
142	nominal	70	0.6	normal	0	0.032	0.993	0.569	0.027	0.014	0.0228	0.61
143	nominal	70	0.6	skewed	0.5	0.239	0.992	0.926	0.227	0.073	0.0228	3.2
144	nominal	70	0.6	skewed	1	0.347	0.983	0.894	0.323	0.111	0.0228	4.87
145	ED	5	0	normal	0	0.363	0.985	0.363	0.222	0.023	0.0228	0.99
146	ED	5	0	skewed	0.5	0.482	0.974	0.513	0.331	0.05	0.0228	2.19
147	ED	5	0	skewed	1	0.511	0.975	0.596	0.38	0.058	0.0228	2.54
148	ED	5	0.2	normal	0	0.327	0.985	0.36	0.206	0.023	0.0228	1.02
149	ED	5	0.2	skewed	0.5	0.454	0.975	0.529	0.324	0.049	0.0228	2.15
150	ED	5	0.2	skewed	1	0.487	0.977	0.624	0.376	0.055	0.0228	2.43
151	ED	5	0.4	normal	0	0.27	0.984	0.368	0.178	0.025	0.0228	1.09
152	ED	5	0.4	skewed	0.5	0.422	0.979	0.59	0.324	0.048	0.0228	2.1
153	ED	5	0.4	skewed	1	0.468	0.984	0.714	0.393	0.052	0.0228	2.3
154	ED	5	0.6	normal	0	0.211	0.983	0.412	0.143	0.027	0.0228	1.2
155	ED	5	0.6	skewed	0.5	0.359	0.984	0.686	0.3	0.046	0.0228	2.01
156	ED	5	0.6	skewed	1	0.41	0.991	0.836	0.376	0.048	0.0228	2.09
157	ED	10	0	normal	0	0.289	0.984	0.29	0.17	0.023	0.0228	0.99
158	ED	10	0	skewed	0.5	0.489	0.958	0.433	0.298	0.07	0.0228	3.07
159	ED	10	0	skewed	1	0.535	0.951	0.513	0.355	0.091	0.0228	3.99
160	ED	10	0.2	normal	0	0.253	0.982	0.295	0.156	0.024	0.0228	1.06
161	ED	10	0.2	skewed	0.5	0.447	0.961	0.465	0.295	0.068	0.0228	2.97
162	ED	10	0.2	skewed	1	0.495	0.958	0.559	0.356	0.085	0.0228	3.71
163	ED	10	0.4	normal	0	0.206	0.981	0.325	0.134	0.027	0.0228	1.19
164	ED	10	0.4	skewed	0.5	0.408	0.969	0.57	0.307	0.064	0.0228	2.83
165	ED	10	0.4	skewed	1	0.468	0.973	0.688	0.384	0.076	0.0228	3.35
166	ED	10	0.6	normal	0	0.16	0.979	0.404	0.106	0.031	0.0228	1.37
167	ED	10	0.6	skewed	0.5	0.342	0.98	0.72	0.287	0.06	0.0228	2.62
168	ED	10	0.6	skewed	1	0.399	0.988	0.853	0.367	0.065	0.0228	2.86
169	ED	20	0	normal	0	0.221	0.982	0.219	0.124	0.023	0.0228	0.99

170	ED	20	0	skewed	0.5	0.511	0.93	0.354	0.265	0.1	0.0228	4.4
171	ED	20	0	skewed	1	0.576	0.907	0.431	0.327	0.146	0.0228	6.39
172	ED	20	0.2	normal	0	0.191	0.98	0.243	0.117	0.025	0.0228	1.1
173	ED	20	0.2	skewed	0.5	0.447	0.939	0.41	0.27	0.094	0.0228	4.14
174	ED	20	0.2	skewed	1	0.504	0.924	0.492	0.331	0.129	0.0228	5.65
175	ED	20	0.4	normal	0	0.158	0.977	0.298	0.101	0.03	0.0228	1.32
176	ED	20	0.4	skewed	0.5	0.402	0.956	0.563	0.296	0.087	0.0228	3.79
177	ED	20	0.4	skewed	1	0.468	0.955	0.655	0.371	0.108	0.0228	4.76
178	ED	20	0.6	normal	0	0.127	0.975	0.422	0.081	0.036	0.0228	1.58
179	ED	20	0.6	skewed	0.5	0.327	0.976	0.762	0.276	0.076	0.0228	3.34
180	ED	20	0.6	skewed	1	0.389	0.983	0.852	0.354	0.087	0.0228	3.82
181	ED	30	0	normal	0	0.191	0.981	0.188	0.105	0.023	0.0228	1
182	ED	30	0	skewed	0.5	0.533	0.907	0.312	0.245	0.125	0.0228	5.48
183	ED	30	0	skewed	1	0.611	0.866	0.388	0.311	0.191	0.0228	8.4
184	ED	30	0.2	normal	0	0.161	0.979	0.218	0.098	0.026	0.0228	1.14
185	ED	30	0.2	skewed	0.5	0.45	0.921	0.383	0.258	0.115	0.0228	5.03
186	ED	30	0.2	skewed	1	0.512	0.896	0.458	0.318	0.163	0.0228	7.14
187	ED	30	0.4	normal	0	0.138	0.975	0.296	0.087	0.032	0.0228	1.42
188	ED	30	0.4	skewed	0.5	0.4	0.946	0.568	0.293	0.102	0.0228	4.47
189	ED	30	0.4	skewed	1	0.472	0.939	0.634	0.365	0.132	0.0228	5.8
190	ED	30	0.6	normal	0	0.112	0.973	0.445	0.07	0.039	0.0228	1.71
191	ED	30	0.6	skewed	0.5	0.32	0.974	0.787	0.271	0.087	0.0228	3.83
192	ED	30	0.6	skewed	1	0.385	0.978	0.845	0.347	0.102	0.0228	4.47
193	ED	50	0	normal	0	0.154	0.98	0.152	0.083	0.023	0.0228	1
194	ED	50	0	skewed	0.5	0.567	0.868	0.264	0.22	0.166	0.0228	7.26
195	ED	50	0	skewed	1	0.667	0.794	0.342	0.292	0.269	0.0228	11.8
196	ED	50	0.2	normal	0	0.135	0.977	0.196	0.082	0.028	0.0228	1.21
197	ED	50	0.2	skewed	0.5	0.456	0.893	0.358	0.247	0.146	0.0228	6.41
198	ED	50	0.2	skewed	1	0.522	0.847	0.418	0.301	0.216	0.0228	9.46
199	ED	50	0.4	normal	0	0.116	0.972	0.303	0.073	0.035	0.0228	1.56
200	ED	50	0.4	skewed	0.5	0.399	0.933	0.578	0.29	0.125	0.0228	5.5
201	ED	50	0.4	skewed	1	0.474	0.914	0.605	0.355	0.167	0.0228	7.31
202	ED	50	0.6	normal	0	0.098	0.97	0.487	0.06	0.043	0.0228	1.9
203	ED	50	0.6	skewed	0.5	0.312	0.971	0.81	0.265	0.103	0.0228	4.51
204	ED	50	0.6	skewed	1	0.383	0.97	0.83	0.338	0.123	0.0228	5.39

205	ED	70	0	normal	0	0.133	0.979	0.128	0.07	0.023	0.0228	1.01
206	ED	70	0	skewed	0.5	0.594	0.834	0.236	0.204	0.2	0.0228	8.76
207	ED	70	0	skewed	1	0.71	0.731	0.315	0.279	0.334	0.0228	14.6
208	ED	70	0.2	normal	0	0.119	0.975	0.187	0.072	0.029	0.0228	1.26
209	ED	70	0.2	skewed	0.5	0.463	0.871	0.349	0.244	0.171	0.0228	7.48
210	ED	70	0.2	skewed	1	0.53	0.807	0.396	0.292	0.256	0.0228	11.2
211	ED	70	0.4	normal	0	0.107	0.97	0.318	0.066	0.038	0.0228	1.66
212	ED	70	0.4	skewed	0.5	0.399	0.923	0.59	0.29	0.142	0.0228	6.24
213	ED	70	0.4	skewed	1	0.478	0.894	0.588	0.35	0.192	0.0228	8.43
214	ED	70	0.6	normal	0	0.09	0.969	0.523	0.055	0.046	0.0228	2.03
215	ED	70	0.6	skewed	0.5	0.308	0.969	0.821	0.262	0.114	0.0228	4.99
216	ED	70	0.6	skewed	1	0.382	0.963	0.818	0.332	0.138	0.0228	6.03
217	ED_shrunk	5	0	normal	0	0.363	0.985	0.363	0.222	0.023	0.0228	0.99
218	ED_shrunk	5	0	skewed	0.5	0.482	0.974	0.513	0.331	0.05	0.0228	2.19
219	ED_shrunk	5	0	skewed	1	0.511	0.975	0.596	0.38	0.058	0.0228	2.54
220	ED_shrunk	5	0.2	normal	0	0.324	0.985	0.365	0.207	0.023	0.0228	0.99
221	ED_shrunk	5	0.2	skewed	0.5	0.453	0.976	0.533	0.325	0.048	0.0228	2.12
222	ED_shrunk	5	0.2	skewed	1	0.486	0.978	0.627	0.377	0.055	0.0228	2.42
223	ED_shrunk	5	0.4	normal	0	0.26	0.985	0.384	0.179	0.023	0.0228	1.01
224	ED_shrunk	5	0.4	skewed	0.5	0.418	0.98	0.607	0.327	0.046	0.0228	2.02
225	ED_shrunk	5	0.4	skewed	1	0.466	0.984	0.725	0.396	0.051	0.0228	2.25
226	ED_shrunk	5	0.6	normal	0	0.194	0.986	0.442	0.141	0.023	0.0228	1.01
227	ED_shrunk	5	0.6	skewed	0.5	0.349	0.987	0.722	0.301	0.042	0.0228	1.84
228	ED_shrunk	5	0.6	skewed	1	0.404	0.993	0.859	0.377	0.045	0.0228	2
229	ED_shrunk	10	0	normal	0	0.289	0.984	0.291	0.17	0.022	0.0228	0.98
230	ED_shrunk	10	0	skewed	0.5	0.489	0.958	0.433	0.298	0.07	0.0228	3.07
231	ED_shrunk	10	0	skewed	1	0.535	0.951	0.513	0.355	0.091	0.0228	3.99
232	ED_shrunk	10	0.2	normal	0	0.247	0.983	0.302	0.156	0.023	0.0228	1.01
233	ED_shrunk	10	0.2	skewed	0.5	0.445	0.962	0.471	0.296	0.066	0.0228	2.91
234	ED_shrunk	10	0.2	skewed	1	0.494	0.959	0.563	0.357	0.084	0.0228	3.68
235	ED_shrunk	10	0.4	normal	0	0.191	0.984	0.345	0.132	0.023	0.0228	1.03
236	ED_shrunk	10	0.4	skewed	0.5	0.401	0.973	0.595	0.31	0.06	0.0228	2.65
237	ED_shrunk	10	0.4	skewed	1	0.466	0.975	0.704	0.387	0.074	0.0228	3.25
238	ED_shrunk	10	0.6	normal	0	0.136	0.986	0.437	0.099	0.024	0.0228	1.04
239	ED_shrunk	10	0.6	skewed	0.5	0.325	0.986	0.769	0.286	0.052	0.0228	2.3

240	ED_shrunk	10	0.6	skewed	1	0.389	0.991	0.884	0.365	0.061	0.0228	2.68
241	ED_shrunk	20	0	normal	0	0.221	0.982	0.22	0.124	0.022	0.0228	0.98
242	ED_shrunk	20	0	skewed	0.5	0.511	0.931	0.354	0.265	0.1	0.0228	4.39
243	ED_shrunk	20	0	skewed	1	0.576	0.907	0.431	0.327	0.145	0.0228	6.38
244	ED_shrunk	20	0.2	normal	0	0.183	0.982	0.25	0.115	0.023	0.0228	1.02
245	ED_shrunk	20	0.2	skewed	0.5	0.443	0.941	0.418	0.273	0.091	0.0228	4.01
246	ED_shrunk	20	0.2	skewed	1	0.503	0.926	0.497	0.333	0.127	0.0228	5.58
247	ED_shrunk	20	0.4	normal	0	0.138	0.982	0.317	0.095	0.024	0.0228	1.06
248	ED_shrunk	20	0.4	skewed	0.5	0.392	0.962	0.594	0.3	0.079	0.0228	3.48
249	ED_shrunk	20	0.4	skewed	1	0.465	0.959	0.675	0.376	0.104	0.0228	4.57
250	ED_shrunk	20	0.6	normal	0	0.097	0.985	0.452	0.07	0.024	0.0228	1.07
251	ED_shrunk	20	0.6	skewed	0.5	0.304	0.985	0.818	0.271	0.064	0.0228	2.82
252	ED_shrunk	20	0.6	skewed	1	0.376	0.988	0.888	0.351	0.08	0.0228	3.51
253	ED_shrunk	30	0	normal	0	0.19	0.981	0.189	0.105	0.023	0.0228	0.99
254	ED_shrunk	30	0	skewed	0.5	0.532	0.908	0.312	0.245	0.125	0.0228	5.46
255	ED_shrunk	30	0	skewed	1	0.611	0.867	0.389	0.311	0.191	0.0228	8.38
256	ED_shrunk	30	0.2	normal	0	0.151	0.981	0.225	0.096	0.024	0.0228	1.03
257	ED_shrunk	30	0.2	skewed	0.5	0.445	0.925	0.392	0.261	0.111	0.0228	4.85
258	ED_shrunk	30	0.2	skewed	1	0.511	0.898	0.464	0.32	0.16	0.0228	7.03
259	ED_shrunk	30	0.4	normal	0	0.115	0.982	0.315	0.08	0.025	0.0228	1.09
260	ED_shrunk	30	0.4	skewed	0.5	0.388	0.956	0.601	0.296	0.092	0.0228	4.05
261	ED_shrunk	30	0.4	skewed	1	0.468	0.946	0.656	0.371	0.126	0.0228	5.55
262	ED_shrunk	30	0.6	normal	0	0.079	0.985	0.473	0.057	0.025	0.0228	1.09
263	ED_shrunk	30	0.6	skewed	0.5	0.293	0.985	0.846	0.264	0.072	0.0228	3.16
264	ED_shrunk	30	0.6	skewed	1	0.37	0.985	0.882	0.343	0.093	0.0228	4.07
265	ED_shrunk	50	0	normal	0	0.152	0.98	0.152	0.083	0.023	0.0228	0.99
266	ED_shrunk	50	0	skewed	0.5	0.565	0.869	0.265	0.22	0.165	0.0228	7.22
267	ED_shrunk	50	0	skewed	1	0.667	0.795	0.342	0.292	0.268	0.0228	11.8
268	ED_shrunk	50	0.2	normal	0	0.122	0.98	0.203	0.078	0.024	0.0228	1.06
269	ED_shrunk	50	0.2	skewed	0.5	0.45	0.899	0.368	0.25	0.14	0.0228	6.14
270	ED_shrunk	50	0.2	skewed	1	0.521	0.852	0.424	0.304	0.212	0.0228	9.28
271	ED_shrunk	50	0.4	normal	0	0.091	0.981	0.321	0.064	0.026	0.0228	1.13
272	ED_shrunk	50	0.4	skewed	0.5	0.385	0.946	0.616	0.293	0.112	0.0228	4.9
273	ED_shrunk	50	0.4	skewed	1	0.47	0.924	0.629	0.361	0.158	0.0228	6.95
274	ED_shrunk	50	0.6	normal	0	0.063	0.985	0.512	0.046	0.025	0.0228	1.11

275	ED_shrunk	50	0.6	skewed	0.5	0.281	0.984	0.87	0.255	0.083	0.0228	3.64
276	ED_shrunk	50	0.6	skewed	1	0.367	0.98	0.87	0.335	0.111	0.0228	4.85
277	ED_shrunk	70	0	normal	0	0.131	0.98	0.129	0.07	0.023	0.0228	0.99
278	ED_shrunk	70	0	skewed	0.5	0.592	0.836	0.237	0.204	0.198	0.0228	8.69
279	ED_shrunk	70	0	skewed	1	0.709	0.732	0.316	0.279	0.332	0.0228	14.6
280	ED_shrunk	70	0.2	normal	0	0.106	0.979	0.194	0.069	0.025	0.0228	1.08
281	ED_shrunk	70	0.2	skewed	0.5	0.457	0.88	0.361	0.247	0.162	0.0228	7.12
282	ED_shrunk	70	0.2	skewed	1	0.529	0.813	0.403	0.295	0.251	0.0228	11.0
283	ED_shrunk	70	0.4	normal	0	0.08	0.98	0.336	0.056	0.026	0.0228	1.15
284	ED_shrunk	70	0.4	skewed	0.5	0.384	0.939	0.63	0.293	0.126	0.0228	5.52
285	ED_shrunk	70	0.4	skewed	1	0.474	0.906	0.613	0.356	0.182	0.0228	7.98
286	ED_shrunk	70	0.6	normal	0	0.055	0.985	0.545	0.04	0.025	0.0228	1.12
287	ED_shrunk	70	0.6	skewed	0.5	0.275	0.984	0.881	0.25	0.091	0.0228	3.97
288	ED_shrunk	70	0.6	skewed	1	0.365	0.975	0.859	0.33	0.123	0.0228	5.4

Note. Classification performance of four rules (any trait > 70, nominal number of factors, effective dimensionality [ED], and shrinkage ED [ED_shrunk]) at cutoff T = 70. Outcomes include sensitivity (true positive rate), specificity (true negative rate), precision (positive predictive value), Jaccard index (overlap between predicted and true cases), caseness rate (proportion classified as cases), target rate (expected prevalence), and inflation (ratio of caseness rate to target rate).

Supplementary Figure S1

Agreement between threshold-based and distance-based case identification (Jaccard index) as a function of trait dimensionality, intercorrelation, and distributional form

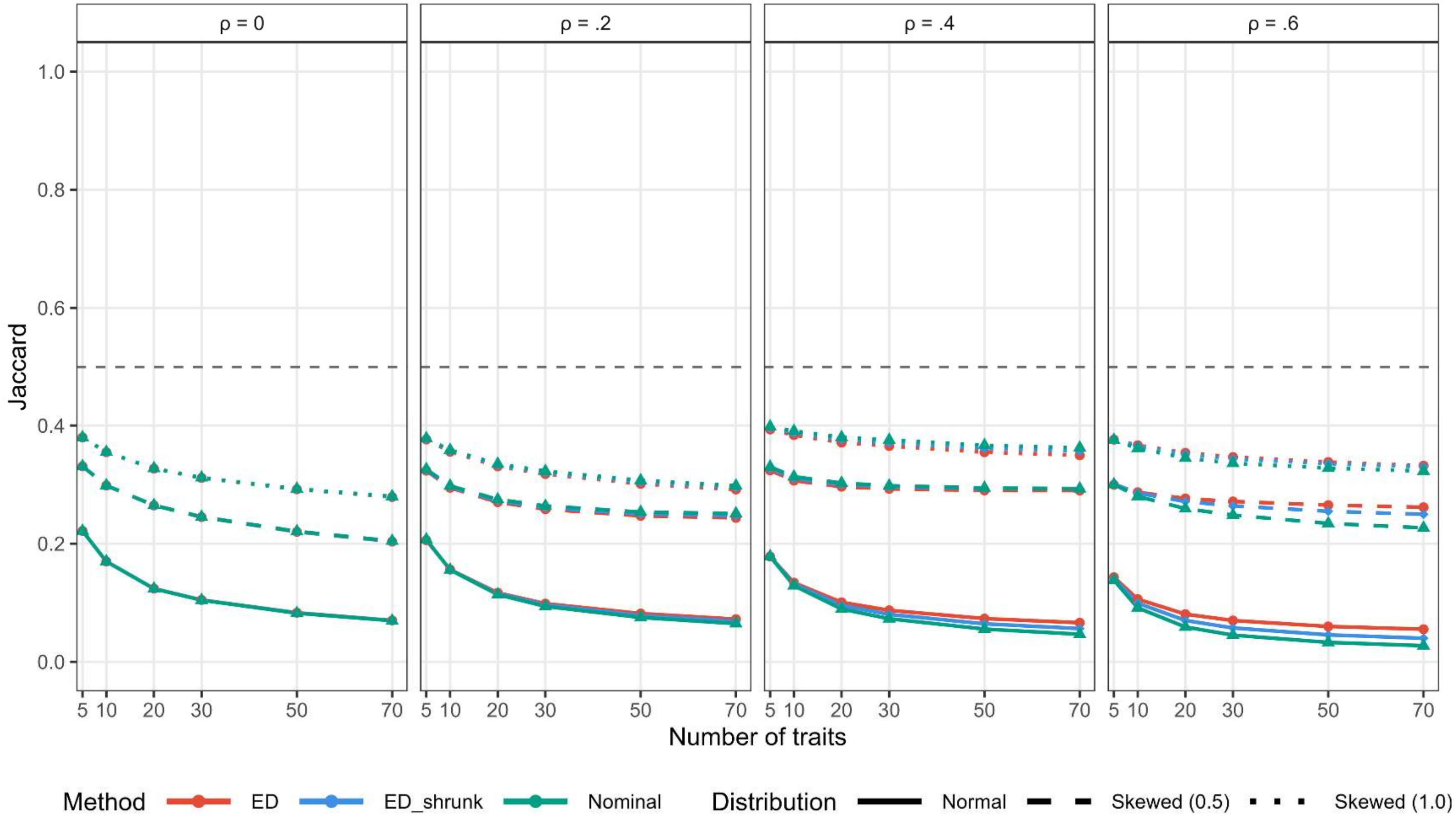

Note. Jaccard index (intersection over union with Mahalanobis distance ground truth) is plotted against the number of traits (k = 5–70) for three adjustment methods (ED, ED_shrunk, Nominal) under varying intercorrelations (ρ = 0, 0.20, 0.40, 0.60) and distributions (Normal, Skewed 0.50, Skewed 1.0). The horizontal dashed line indicates Jaccard = 0.50.

Supplementary Figure S2

Sensitivity and specificity of dimensionality-adjusted cutoff methods as a function of trait dimensionality, intercorrelation, and distributional form

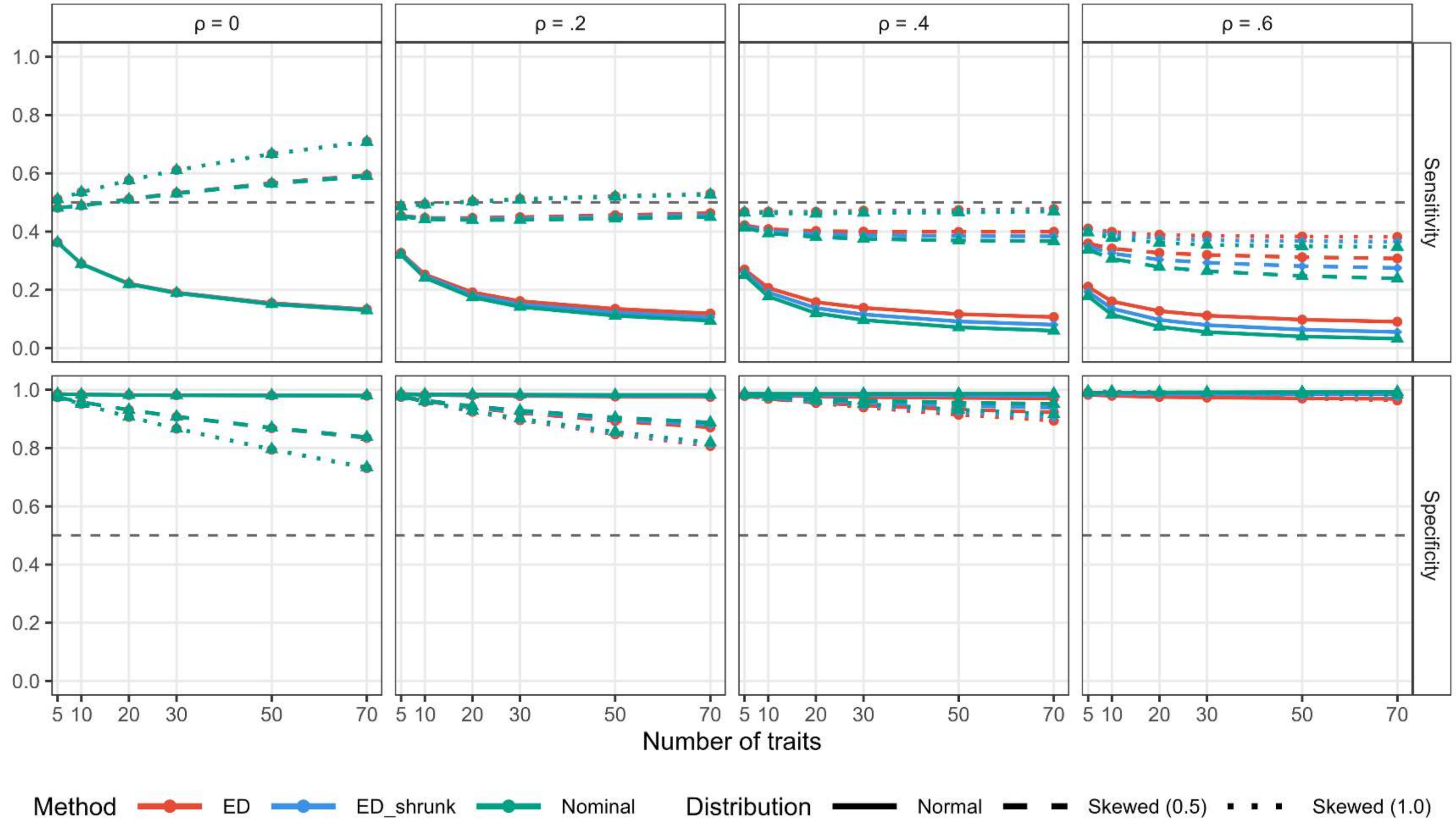

Note. Sensitivity (top panels) and specificity (bottom panels) are plotted against the number of traits (k = 5–70) for three adjustment methods (ED, ED_shrunk, Nominal) under varying intercorrelations (ρ = 0, 0.20, 0.40, 0.60) and distributions (Normal, Skewed 0.50, Skewed 1.0). The horizontal dashed line indicates 0.50 for reference.
